# LED red light therapy for relief of primary dysmenorrhea: An open, single-group target value clinical trial

**DOI:** 10.1371/journal.pone.0356618

**Published:** 2026-09-01

**Authors:** Bin Yi, Dandan Wang, Ling Wei, Jun He, Qian Yu, Lei Wu, Yilin Wang, Yajuan Wang, Nan Song, Li Guo

**Affiliations:** 1 Beijing Charmwin Light Medical Technology Co., Ltd., Beijing, China; 2 Department of Gynecology and Obstetrics, The First Affiliated Hospital of Baotou Medical College, Baotou, China; 3 Department of Gynecology, Hebei PetroChina Central Hospital, Langfang, China; 4 Department of Dermatology, Chinese PLA General Hospital, Beijing, China; Sapienza University of Rome, ITALY

## Abstract

**Objective:**

To evaluate the efficacy and safety of LED red light therapy in alleviating primary dysmenorrhea.

**Methods:**

A total of 119 patients with primary dysmenorrhea were enrolled in 2022. The intervention was LED red light therapy administered 7 days before menstruation. Clinical outcomes were assessed after three menstrual cycles.

**Results:**

After three cycles of treatment, the mean reduction rate in the Visual Analog Scale (VAS) score was 63.62%. The overall effective rate of the device was 98.32%. The total score of the COX Menstrual Symptom Scale decreased by 72.93%, with specific reductions of 72.55% in symptom severity and 73.26% in pain duration. No severe adverse events were reported during the trial. Only one adverse event (0.84%) was considered device-related.

**Conclusion:**

LED red light therapy is safe and effective for the treatment of primary dysmenorrhea.

**Trial registration:**

ChiCTR2600116460.

## Introduction

Primary dysmenorrhea (PD) ranks among the most prevalent gynecological disorders in women of reproductive age, with a reported incidence ranging from 50% to 90%. It is characterized by cramping pain during menstruation, frequently accompanied by systemic symptoms such as fatigue and nausea, which significantly impair the quality of life and cause productivity loss [1]. The underlying pathophysiology is primarily associated with excessive secretion of endometrial prostaglandins during the menstrual cycle. Specifically, an imbalance between prostaglandin F2α (PGF2α) and prostaglandin E2 (PGE2) levels triggers spasmodic contractions of uterine smooth muscle, localized ischemia, and increased neural sensitivity.

Current first-line clinical management relies on non-steroidal anti-inflammatory drugs (NSAIDs) and hormonal therapies. However, long-term NSAID use may lead to adverse effects including gastrointestinal injury and renal dysfunction, while oral contraceptives carry potential risks such as thrombosis, breakthrough bleeding, and endocrine disturbances [1,2]. Of particular concern is that approximately 20% to 25% of patients exhibit an inadequate response to conventional pharmacological treatments or cannot tolerate their side effects. This highlights an urgent need for the development of non-pharmacological, non-invasive, safe, and effective alternative therapies [2].

In recent years, physical therapy has gained increasing prominence in the management of PD. Among various modalities, LED red light therapy—based on the principle of photobiomodulation (PBM)—has emerged as a research focus in pain management due to its deep tissue penetration, non-invasive nature, and dual regulatory capacity in alleviating inflammation and pain. PBM therapy has been widely reported in the treatment of wounds [3], musculoskeletal disorders, and neuropathic symptoms [4], promoting tissue healing [5] and pain relief [6] thereby attracting significant attention in biomedical research.

Owing to advantages such as high efficiency, long lifespan, flexible wavelength selection, compact size, and low power consumption, LEDs have been widely used in PBM applications. Red light therapy typically employs low-intensity light sources within the wavelength range of 630–670 nm. The emitted energy penetrates subcutaneous tissues and is absorbed by mitochondrial chromophores, thereby regulating cellular metabolism to exert therapeutic effects. Previous studies [7] have confirmed that abdominal irradiation with red light (610 nm, 1.8 mW/cm^2^, 20 min) significantly reduces dysmenorrhea scores. In a study by Zhu et al. [8], LED-based irradiation (630 nm, 2.5 mW/cm^2^, 3 J/cm^2^) applied to acupoints CV4 and CV6 for 20 minutes daily demonstrated pain relief effects comparable to those of combined oral contraceptives (DSG/EE). These foundational studies have established a scientific basis for optical therapy in PD. The present study utilized an LED red light therapy device (wavelength: 640 nm) for the treatment of patients with PD. The findings are reported as follows.

## Materials and methods

### Study design

This was an open, single-group target-value clinical trial. The unit of analysis for evaluating intervention efficacy was the individual participant. No group allocation was performed, and all participants received the same LED red light therapy. The trial adhered to the Declaration of Helsinki, with strict participant screening to minimize bias. The target value for evaluating the effectiveness of the LED red light therapy device was preset to 80%, based on the assumption that the experimental device would achieve a 90% effective rate. Combined with the preset effective rate and target value, 120 participants were enrolled in this study. This clinical trial was compliant with standard guidelines and registered at ChiCTR.org (ChiCTR2600116460). Registration was performed retrospectively after enrolment started because we were unaware of ICMJE trial registration rules at study initiation and only learned the requirement when preparing this manuscript. The authors confirm that all ongoing and related trials for this intervention are registered. The study protocol adhered to the ethical principles of the Declaration of Helsinki and was approved by the Clinical Trial Ethics Committees of The First Affiliated Hospital of Baotou Medical College, Inner Mongolia University of Science and Technology (Approval No. 2022 Ethics Review (Supplement) No. (031)) and Hebei PetroChina Central Hospital (IRB2021-115-02).

### Sample size calculation


$$\begin{array}{c} n_{T}=\frac{\left[Z_{\text{1}-\alpha }\sqrt{P_{\text{0}}(\text{1}-P_{\text{0}})}+Z_{\text{1}-\beta }\sqrt{{P_{T}(\text{1}-P_{T})}^{\text{2}}}\right]}{(P_{T}-P_{\text{0}}{)}^{\text{2}}} \end{array}$$

The key parameters were set as follows:

α, significance level: α = 0.025;

1-β, power of test: β = 0.20;

Assume that the efficiency rate of the test equipment (${\text{P}}_{\text{T}}$) is 90%, and set the target value (${\text{P}}_{\text{0}}$) as 80%.

After calculation, the sample size is 107 cases. Considering a 10% dropout rate and clinical operation requirements, the required sample size is 120 cases.

### Study patients

**Fig 1:**
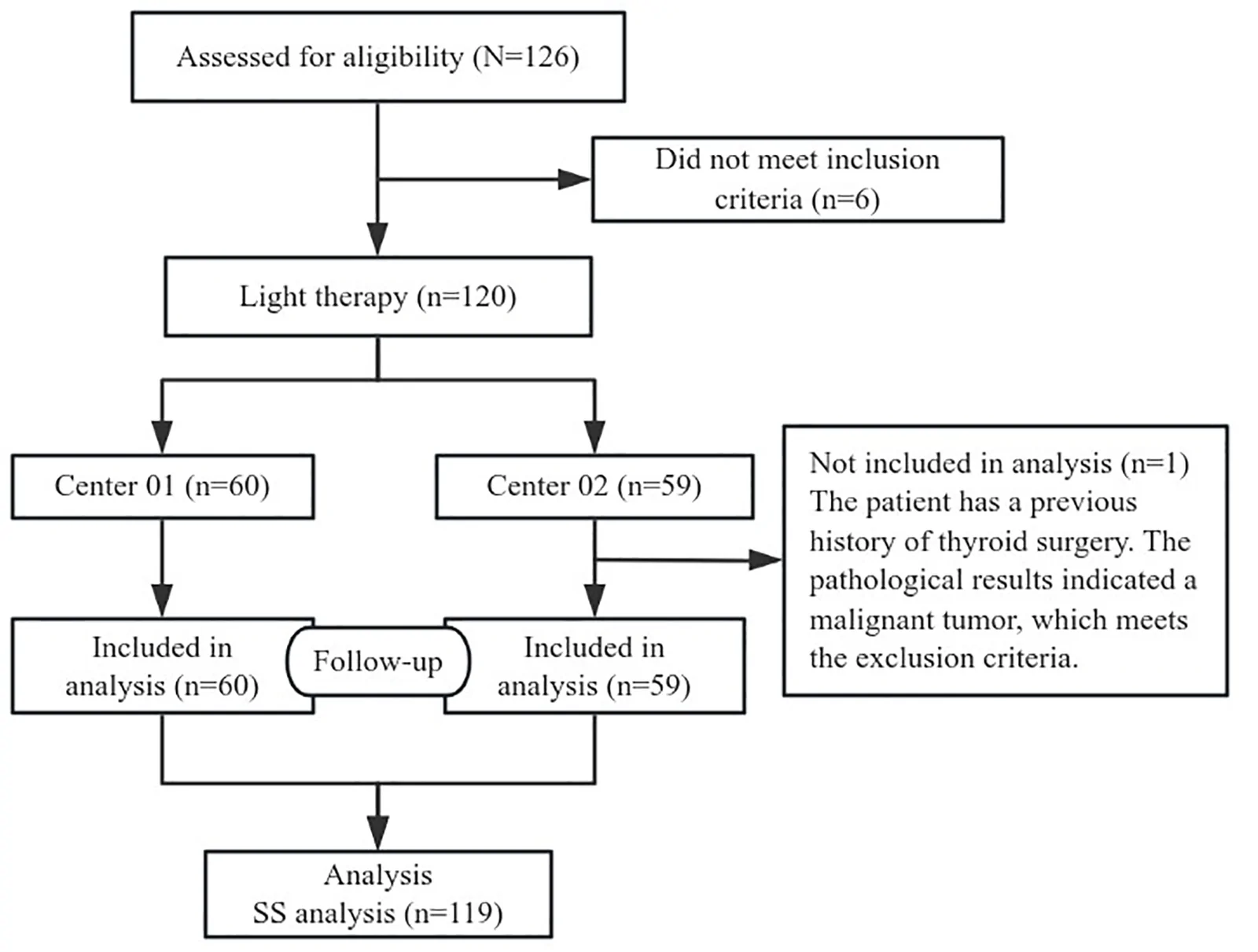
The study flow chart.

Participants were recruited from April to June 2022. Participants were recruited via public advertisements and screened according to pre-specified inclusion and exclusion criteria. The recruitment sites of this study were the First Affiliated Hospital of Baotou Medical College (Center 01) and Hebei PetroChina Central Hospital (Center 02). Research physicians were responsible for screening and registration to ensure that the recruited population met the study design requirements. All subjects signed a written informed consent form, and minor participants obtained informed consent from their legal guardians. A total of 120 patients with PD were enrolled in this study. One patient dropped out during treatment, and 119 patients completed the treatment, as illustrated in Fig 1. Participants were aged 14–34 years, with a mean age of 27 ± 3.93 years.

Eligible participants were required to meet all of the following criteria: 1) female patients aged 13–35 years (inclusive); 2) diagnosis of primary dysmenorrhea; 3) regular menstrual cycles (28 ± 7 days); 4) self-reported average pain visual analogue scale (VAS) score of ≥ 4 during the most recent two menstrual cycles; and 5) voluntary participation with written informed consent provided by the patient and/or legal guardian.

The exclusion criteria were as follows: diagnosis of secondary dysmenorrhea confirmed by gynecological examination and abdominal ultrasonography due to gynecological conditions such as endometriosis, pelvic inflammatory disease, or adenomyosis, or other organic pathologies; use of oral contraceptives and/or centrally acting medications and/or receipt of physical therapy for dysmenorrhea within the past month; use of analgesic medications within 24 hours prior to evaluation; presence of skin ulcers, infections, or other abnormalities at the irradiation site; severe psychiatric or other disorders impairing the ability to accurately complete VAS pain assessments or objectively describe symptoms; history of phototherapy allergy, malignant tumors, systemic lupus erythematosus, hemorrhagic/hemolytic diseases; pregnancy, lactation, or planned pregnancy within 3 months after enrollment; participation in other clinical trials (involving medical devices or pharmaceuticals) within the past month; other conditions deemed by the investigators to render the participant unsuitable for inclusion, such as anticipated poor compliance.

### Study treatments

**Fig 2:**
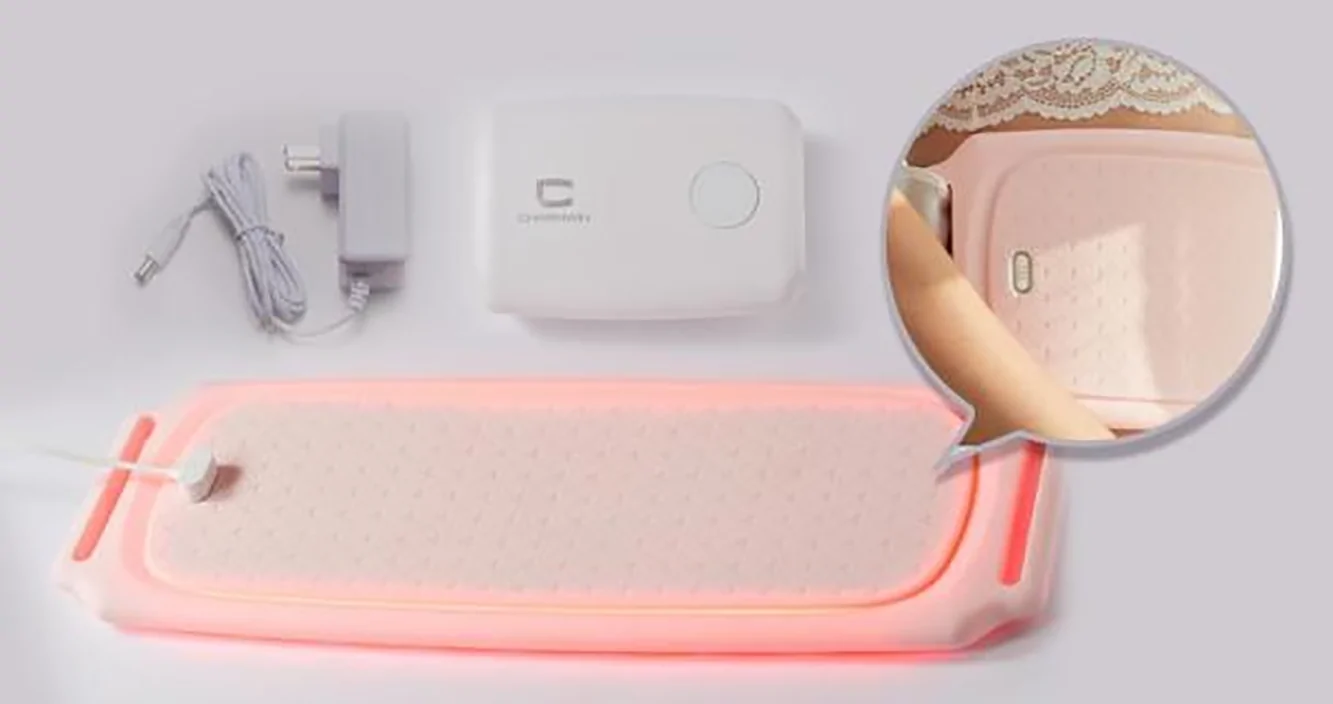
LED red light therapy device. Reprinted from company product images under a CC BY license, with permission from Beijing Charmwin Light Medical Technology Co., Ltd., original copyright 2026.

The experimental group used the LED red light therapy device (Beijing Charmwin Light Medical Technology Co., Ltd., CY-1002C, 640 nm ± 10 nm, 25 mW/cm^2^, 220 VAC, 50 W), as illustrated in Fig 2. Researchers provided the LED red light therapy device to participants, who used it independently at home after standardized training. The intervention was initiated 7 days prior to the anticipated onset of menstruation. Each session lasted 30 minutes and was administered once daily. Treatment was suspended during menstruation. Follow-up assessments were conducted over three consecutive menstrual cycles after the initiation of treatment to evaluate improvements in dysmenorrhea symptoms. In principle, all routine analgesics were prohibited during the entire study period.

### Parameters and assessments

#### Efficacy observation indicators.

Pain intensity was measured using the Visual Analogue Scale (VAS; 0–10 points) [8], where higher scores indicate more severe pain. The pain improvement rate was classified into three grades: marked effectiveness, effective, and ineffective. The overall response rate was defined as the proportion of patients achieving a “marked effectiveness” or “effective” outcome. The severity and duration of dysmenorrhea symptoms were assessed using the COX Dysmenorrhea Symptom Scale (COX) [9]. Symptom severity was graded as none, mild discomfort, moderate discomfort, severe discomfort, and extreme discomfort. The duration was graded as none, less than 3 hours, 3–7 hours, 7–24 hours, and more than 24 hours. The scores were assigned from low to high as 0, 1, 2, 3, and 4 points.

### Criterion of therapeutical effect

The rate of reduction in VAS score was calculated as follows:


$$\begin{array}{cc} \text{Reduction Rate }(\%)\;= & \;[(\text{Screening Phase VAS Score }-\text{ Post}-\text{treatment VAS Score})/\\ & \text{ Screening Phase VAS Score}]\;\times \text{ 100}\%. \end{array}$$

Therapeutic outcomes were categorized based on the VAS score reduction rate: Marked Effectiveness: Reduction rate ≥ 70%; Effective: Reduction rate between 30% and 69%; Ineffective: Reduction rate ≤ 29%

The overall response rate was defined as the proportion of patients achieving either “Marked Effectiveness” or “Effective” outcomes, calculated as:


$$\begin{array}{c} Overall\;Response\;Rate\;(\%)\;=\;[(Number\;of\;``Marked\;Effectiveness"\;Cases\;\\ +\;Number\;of\;``Effective"\;Cases)/\;Total\;Number\;of\;Cases]\;\times \;100\%. \end{array}$$

### Patient Satisfaction with the Product

Patient experience with the product was assessed based on subjective feedback following its use. The experience was rated using a 5-point scale: 1) Very inconvenient, dissatisfied; 2) Inconvenient, dissatisfied; 3) Moderately portable, moderately satisfied; 4) Portable, satisfied; 5) Very portable, very satisfied. For analysis, ratings of 1-3 were defined as a “poor” experience, while ratings of 4-5 were defined as a “good” experience.

### Statistical analysis

Statistical analyses were conducted using SAS 9.4. For the primary efficacy endpoint, the response rate was presented as frequency and percentage, with its two-sided 95% confidence interval derived from the Clopper-Pearson exact method. For the continuous efficacy variable, the reduction rate in the VAS score was summarized by descriptive statistics including mean, standard deviation, median, min, max, Q1, and Q3.

## Results

### Baseline characteristics

A total of 120 participants were enrolled in the current study, of whom 119 completed all the study process. There was one case of loss to follow-up. As shown in Table 1, the baseline characteristics of the participants are presented. No participants received rescue analgesic medication throughout the trial.

**Table 1:** Basal characteristics of the participants.

Characteristics	N (Missing)	Mean (SD)	Median	Q1, Q3	Min, Max
Age, years	119 (0)	27.00 (3.93)	27.00	24.00, 30.00	14.00, 34.00
Height, cm	119 (0)	164.26 (4.94)	165.00	160.00, 168.00	152.00, 176.00
Weight, kg	119 (0)	58.30 (10.90)	57.00	51.00, 63.00	42.00, 105.00
Self-assessment scores of the VAS scale over the past two menstrual cycles	116 (3)	6.22 (0.99)	6.00	5.50, 7.00	4.00, 9.00

### Pain improvement rate

**Fig 3:**
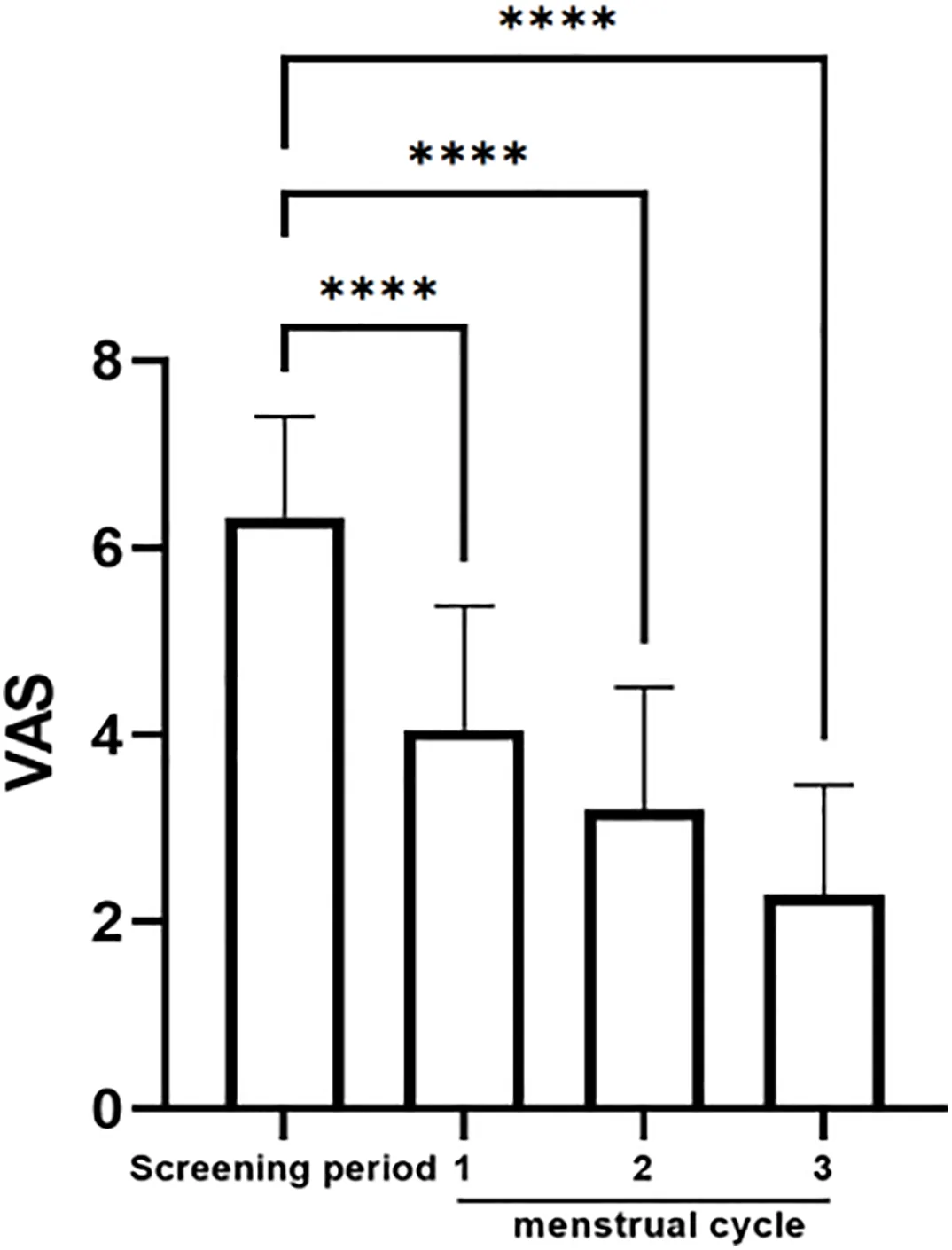
VAS Scores.

The VAS pain scores for dysmenorrhea, summarized in Table 2, were recorded during the screening period and after the first, second, and third menstrual cycles following enrollment. The mean scores were 6.33, 4.06, 3.20, and 2.29, respectively, demonstrating a significant downward trend and indicating marked symptomatic relief (Fig 3). The mean reduction rate was 63.62%.

**Table 2:** Summary of VAS Scores.

Stage	N (Missing)	Mean (SD)	Median	Q1, Q3	Min, Max
Screening period	119 (0)	6.33 (1.07)	6.00	6.00, 7.00	4.00, 9.00
The first menstrual period	119 (0)	4.06 (1.32)	4.00	3.00, 5.00	1.00, 8.00
The second menstrual period	119 (0)	3.20 (1.31)	3.00	2.00, 4.00	0.50, 7.00
The third menstrual period	119 (0)	2.29 (1.17)	2.00	1.00, 3.00	0.00, 7.00

### Main therapeutic effect

The main efficacy result evaluation analysis is shown in Table 3. The effective rates of the subjects during the first, second and third menstrual cycles after enrollment were 71.43%, 94.12%, 98.32%, respectively. After three cycles of treatment, the overall effective rate of the device reached 98.32%.

**Table 3:** The main efficacy result evaluation analysis.

Stage	N(Missing)	Marked Effectiveness(%)	Effective(%)	Ineffective(%)	Overall Response Rate (%)	95% CI (Clopper-Pearson)
The first menstrual period	119 (0)	5 (4.20%)	80 (67.23%)	34 (28.57%)	85 (71.43%)	62.43% ~ 79.33%
The second menstrual period	119 (0)	17 (14.29%)	95 (79.83%)	7 (5.88%)	112 (94.12%)	88.26% ~ 97.60%
The third menstrual period	119 (0)	47 (39.50%)	70 (58.82%)	2 (1.68%)	117 (98.32%)	94.06% ~ 99.80%

### COX

**Fig 4:**
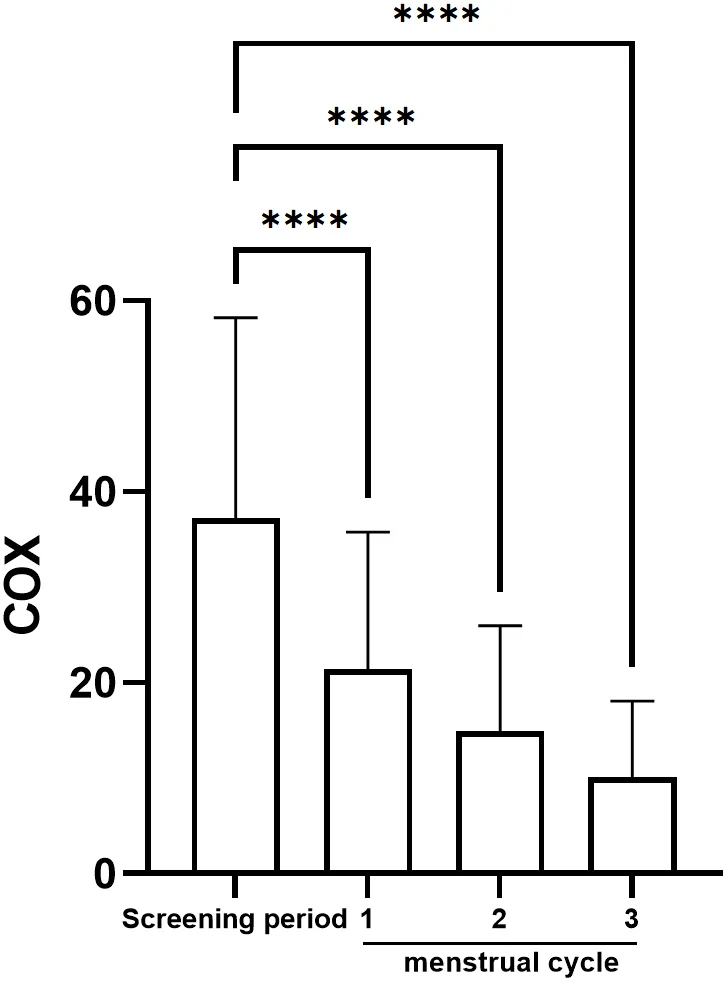
The mean total score of the COX Menstrual Symptom Scale.

Scores from the COX Menstrual Symptom Scale are presented in Table 4. The mean total scores recorded at screening and after the first, second, and third menstrual cycles were 37.24, 21.41, 14.92, and 10.08, respectively (Fig 4). The mean symptom severity scores for these intervals were 17.16, 10.05, 6.94, and 4.71, while the mean symptom duration scores were 20.08, 11.36, 7.98, and 5.37. These results indicated a consistent and significant reduction in dysmenorrhea symptoms. After three cycles of treatment, the total score of the COX Menstrual Symptom Scale decreased by 72.93%, including a 72.55% reduction in symptom severity and a 73.26% reduction in pain duration.

**Table 4:** Scores from the COX Menstrual Symptom Scale.

Stage	Scores	N (Missing)	Mean (SD)	Median	Q1, Q3	Min, Max
Screening period	Total	119 (0)	37.24 (20.96)	33.00	22.00, 48.00	8.00, 108.00
	Symptom Severity	119 (0)	17.16 (9.90)	15.00	10.00, 22.00	4.00, 54.00
	Symptom Duration	119 (0)	20.08 (11.82)	17.00	11.00, 27.00	4.00, 54.00
The first menstrual period	Total	119 (0)	21.41 (14.37)	18.00	12.00, 29.00	0.00, 69.00
	Symptom Severity	119 (0)	10.05 (6.59)	8.00	5.00, 13.00	0.00, 32.00
	Symptom Duration	119 (0)	11.36 (8.25)	9.00	6.00, 15.00	0.00, 42.00
The second menstrual period	Total	119 (0)	14.92 (11.06)	12.00	6.00, 22.00	0.00, 53.00
	Symptom Severity	119 (0)	6.94 (4.92)	6.00	3.00, 10.00	0.00, 27.00
	Symptom Duration	119 (0)	7.98 (6.47)	6.00	3.00, 12.00	0.00, 35.00
The third menstrual period	Total	119 (0)	10.08 (7.97)	8.00	4.00, 13.00	0.00, 44.00
	Symptom Severity	119 (0)	4.71 (3.58)	4.00	2.00, 6.00	0.00, 17.00
	Symptom Duration	119 (0)	5.37 (4.64)	4.00	2.00, 7.00	0.00, 29.00

### Patient satisfaction with the product

As shown in Table 5, the proportion of participants who reported a “good” experience with the investigational device was 84.48%, 85.34%, and 87.29% after the first, second, and third menstrual cycles, respectively. The consistently high ratings across all three cycles demonstrated a high level of user acceptance and satisfaction.

**Table 5:** Patient Satisfaction with the Product.

Stage	Experience	Total(%)	95% CI (Wilson)
The first menstrual period	“Poor” experience	18 (15.52%)	(9.98%, 23.10%)
	“Good” experience	98 (84.48%)	(76.90%, 90.02%)
	N(Missing)	116 (3)	–
The second menstrual period	“Poor” experience	17 (14.66%)	(9.27%, 22.49%)
	“Good” experience	99 (85.34%)	(77.51%, 90.73%)
	N(Missing)	116 (3)	–
The third menstrual period	“Poor” experience	15 (12.71%)	(7.82%, 20.01%)
	“Good” experience	103 (87.29%)	(79.99%, 92.18%)
	N(Missing)	118 (1)	–

### Safety evaluation

Adverse events (AEs) occurred in 26.89% (32/119) of participants. All events were mild, with no serious AEs reported. Notably, only a single event (0.84%, 1/119) was considered device-related. The most commonly reported AEs were diarrhea and menorrhagia.

## Discussion

PD is a prevalent gynecological disorder that significantly compromises the quality of life among women of reproductive age. The exact etiology of PD remains unclear, and effective therapeutic options are currently limited. Consequently, identifying safe and effective approaches to alleviate PD represents a critical direction in clinical research. This study employed an open, single-group target-value design to evaluate a home-based therapeutic intervention with the aim of establishing a reliable treatment modality that offers confirmed efficacy and a favorable safety profile.

The findings of this study demonstrate that red light therapy exhibits significant efficacy in alleviating dysmenorrhea symptoms and offers distinct advantages over conventional treatments. Red light therapy exerts its effects through photobiomodulation, promoting tissue repair and mitigating inflammatory processes [6]. It is noteworthy that the therapeutic benefits of red light therapy manifest in a time-dependent manner. In contrast to oral contraceptives, which act rapidly, red light therapy typically requires regular administration for 1–3 months to achieve stable therapeutic outcomes. This difference may be attributed to their fundamentally distinct mechanisms of action: pharmacological interventions rapidly modulate endometrial prostaglandin levels via hormonal regulation, whereas red light therapy induces gradual cellular-level adaptations to achieve sustained improvement. Once established, the therapeutic benefits of red light therapy can often be maintained over the long term, offering a notable advantage over drug-based regimens that require continuous administration. Given the single-group and open-label nature of this trial, placebo effects and psychological factors may have influenced the final outcomes. We cannot rule out the contribution of such factors to symptom relief. While the present results are encouraging, we recognize that it is difficult to quantitatively separate the true efficacy of LED red light therapy from placebo responses under the current study design.

The molecular mechanisms underlying red light therapy for dysmenorrhea involve multi-level biological processes. At the cellular level, red light wavelengths are absorbed by mitochondrial cytochrome c oxidase, leading to an increased mitochondrial membrane potential and enhanced ATP synthesis, thereby elevating cellular energy metabolism [1]. This process not only augments the functional activity of uterine cells but also modulates gene expression through the activation of multiple signaling pathways, resulting in anti-inflammatory, antioxidant, and cytoprotective effects.

In terms of inflammatory regulation, red light therapy significantly reduces the concentrations of pain-related substances such as prostaglandin PGF2α and endothelin (ET), while simultaneously modulating various inflammatory factors [1]. Studies have shown that red light irradiation decreases local serotonin levels, thereby alleviating pain perception. Furthermore, red light inhibits the activation of the NF-κB signaling pathway, leading to reduced release of pro-inflammatory cytokines including TNF-α and IL-1β [10], which may represent one of the key mechanisms underlying its efficacy in ameliorating dysmenorrhea.

Compared with NSAIDs, red light therapy eliminates the risks of gastrointestinal injury and nephrotoxicity. In contrast to oral contraceptives, it causes no hormone-related adverse effects, making it more suitable for women with contraindications to oral contraceptives or hypersensitivity to hormonal therapy. Studies have shown that although oral contraceptives produce faster initial effects, no significant difference in overall efficacy has been observed between red light therapy and oral contraceptives after two treatment cycles [10].

Notably, red light therapy can exert complementary effects when combined with conventional treatments. For acute and severe dysmenorrhea, pharmacological agents may be prioritized for rapid symptom control, whereas red light therapy serves as a long-term management and preventive strategy to reduce drug dependency and associated adverse effects. Such an integrated therapeutic approach may represent an optimized strategy for the comprehensive management of dysmenorrhea.

Despite the promising evidence supporting the efficacy of red light therapy for dysmenorrhea provided by this study, several limitations should be acknowledged. First, this is an unblinded single-group target trial without a placebo control group. Placebo effects may interfere with therapeutic outcomes, and we cannot differentiate the real treatment effect from psychological responses, reducing the certainty of our efficacy results. Second, the limited number of included studies may affect the robustness of the findings. Third, heterogeneity in red light treatment parameters—such as wavelength, energy density, and irradiation duration—across different studies complicates direct comparisons. Furthermore, no consensus currently exists regarding the optimal treatment protocol, including single-session duration, treatment frequency, and overall course, necessitating further research to refine these parameters.

The development and application of home-based red light devices can substantially enhance treatment accessibility and patient compliance. With the advancement of wearable photoelectronic technology, the design of convenient and safe home-use red light therapy devices is expected to revolutionize the management paradigm for dysmenorrhea.

## Supporting information

S1 Protocol Medical Device Clinical Trial Protocol-English. (PDF)

S2 Protocol Medical Device Clinical Trial Protocol. (PDF)

S3 Protocol Protocol CC BY4.0 Permission. (PDF)

S1 Checklist Note of the TREND Statement Checklist. (DOCX)

S2 Checklist TREND Statement Checklist. (PDF)
