# Supplementary material for: LED red light therapy for relief of primary dysmenorrhea: An open, single-group target value clinical trial
Source: PLoS One. 2026 Sep 1;21(9):e0356618. doi: 10.1371/journal.pone.0356618 (PMC13533393; doi:10.1371/journal.pone.0356618)
Supplement: Supplementary file 1 [file pone.0356618.s001.pdf]

# Medical Device Clinical Trial Protocol

Proposal Number: CYGL-2021-02

## Open-label, single-arm evaluation of clinical safety and efficacy of LED red light therapy device
## Group Target Value Clinical Trial

Name of the medical device under trial: LED Red Light Therapy Device

Model specifications: CY1002C

Management category of experimental medical devices: Class II

Class III medical devices that require clinical trial approval □ Yes ☑ No

Similarproducts in China are □ Yes ☑ No

Version and date of the solution: v3.0 / March 14,2022

Clinical trial institution: The First Affiliated Hospital of Baotou Medical College, Inner Mongolia University of Science and Technology

Researcher: Li Guo

Sponsor: Beijing Charmwin Light Medical Technology Co., Ltd.

Agent: Not applicable

filling explanation：

1. For multicenter clinical trials, only the lead institution should be listed on the cover of the clinical trial protocol, while other participating institutions should be specified in the protocol content.

2. For multicenter clinical trials, the investigator on the cover page should fill in the coordinating investigator.

# Catalogue


Scenario Summary....4
I. Sponsor Information....7
II. List of All Clinical Trial Institutions and Investigators in the Multicenter Clinical Trial....7
III. Objectives and Content of Clinical Trials....7
(I) Objective....7
(II) Content....7
IV. Background Information on Clinical Trials....7
V. Product Features, Structural Composition, Working Principle, Mechanism of Action, and Test Scope10
(I) Product Features....10
(II) Product Structure Composition, Working Principle, and Mechanism of Action....10
(III) Scope of the trial....10
VI. Scope of Product Indications, Contraindications, and Precautions....10
(I) Scope of Application....10
(II) Contraindications....10
(III) Precautions....11
VII. Overall Design....11
(I) Experimental Design....11
(II) Trial Procedure....18
(III) Monitoring Plan....20
VIII. Statistical Considerations....21
IX. Data Management....27
(1) Database establishment and management....27
(2) Monitoring of raw data....27
(3) Data Entry and Modification....27
(4) Data Verification....27
(5) Database locking....27
(6) Data archiving....28
X. Feasibility Analysis....28
(I) Probability Analysis of Success....28
(II) Analysis of Failure Probability....28
XI. Quality Control in Clinical Trials....28
XII. Ethical Issues in Clinical Trials and Informed Consent....29
(I) Ethical considerations....29
(II) Approval of the trial protocol....29
(III) Informed consent process and informed consent form text....30
XIII. Provisions on Reporting Adverse Events and Device Defects....30
(I) Adverse Events....30
(II) Serious Adverse Event (SAE)....31
(III) Instrumental Defects....32
(IV) Reporting Procedures and Contact Information....32
XIV. Deviations from Clinical Trial Protocols and Regulations for Clinical Trial Protocol Amendments33
XV. Direct access to source data and files....33
XVI. Finance and Insurance....33
XVII. Contents to be included in clinical trial reports....33
XVIII. Responsibilities Assumed by All Parties....35
1. Sponsor Responsibilities....35
2. Responsibilities of Clinical Trial Institutions and Investigators....36

## Scenario Summary

**Basic Information of Medical Devices:**

Registered category: Class II

Device name: LED Red Light Therapy Device

Model: CY-1002C

Scope of application: Primary dysmenorrhea

Sponsor: Beijing Charmwin Light Medical Technology Co., Ltd.

**Test topic:**

An open, single-group target value clinical trial to evaluate the safety and efficacy of LED red light therapy devices in clinical application

**purpose of research：**

To evaluate the safety and efficacy of LED red light therapy devices in alleviating primary dysmenorrhea

**Study population and sample size:**

It is planned to select subjects with diagnosed primary dysmenorrhea for the study.

**Sample size estimation**

Sample size calculation formula:

$$nT=\frac{[Z_{1-\alpha}\sqrt{P_0(1-P_0)}+Z_{1-\beta}\sqrt{P_T(1-P_T)}]^2}{(P_T-P_0)^2}$$

Parameter settings:

1. α, significance level: In this trial, α=0.025 (unilateral) was taken;

2. 1-β, degree of control: In this experiment, β=0.20;

3. P0 and PT: Assuming the effective rate (PT) of the test equipment is 90%, set the target value (P0) to 80%. Through calculation, the sample size of 107 cases, 10% consider the fall off and clinical operation, the required sample size of 120 cases.

**experiment design**

**system design：**

This trial adopts an open, single-group target value design.

**Validity evaluation index:**

Primary efficacy endpoint: Response rate: The therapeutic effect was statistically analyzed based on the Visual Analogue Scale (VAS)

(Evaluation time node: during the third menstrual cycle after enrollment)

Markedly effective: VAS score reduction rate ≥70%;

Effective: VAS score reduction rate of 30%-69%;

Ineffective: VAS score reduction rate ≤29%.

The rate of VAS score decline = ((VAS score at screening-VAS score after treatment) / VAS score at screening) × 100%.

Effective = Markedly effective + Effective.

**Secondary efficacy indicators:**

1. COX Dysmenorrhea Symptom Scale score;
2. Subject usage experience evaluation (calculated by the 5-order scoring method).
    1) This is extremely inconvenient and unsatisfactory.
    2) Inconvenience, dissatisfaction,
    3) Generally portable, generally satisfactory,
    4) Portable and user-friendly,
    5) Highly portable and very satisfactory.

Items 1) to 3) are defined as indicating poor user experience, while items 4) to 5) are defined as indicating good user experience.

3. VAS scores during the first, second, and third menstrual periods after enrollment;
4. Effective rate during the first and second menstrual cycles after subject enrollment.

**Safety evaluation indicators:**

1. Vital signs

Respiration, pulse, blood pressure, body temperature;

2. Adverse Events

Record any adverse events occurring during the trial, including the name of the adverse event, time of occurrence, duration, severity, and its relationship with the device.

3. Mechanical defects

Refers to unreasonable risks that may endanger human health and life safety during clinical trials when medical devices are used under normal conditions, such as labeling errors, quality issues, malfunctions, etc.

| |
|---|
| **Enrollment/exclusion criteria for subjects:**<br>**Inclusion criteria:**<br>1) Female individuals aged 13–35 years (inclusive of 13 and 35 years old);<br>2) Diagnosed with primary dysmenorrhea;<br>3) Regular menstrual cycle: 28±7 days;<br>4) Individuals who self-reported a mean VAS pain score ≥4 points during the last two menstrual cycles;<br>5) Voluntary participation in the trial, with informed consent signed by the subject and/or one guardian.<br>**Exclusion criteria:**<br>1. Secondary dysmenorrhea confirmed by gynecological examination and abdominal ultrasound due to gynecological diseases such as endometriosis, pelvic inflammatory disease, adenomyosis, or other conditions;<br>2. Screening for individuals who have taken oral contraceptives and/or medications acting on the central nervous system and/or undergone physical therapy related to dysmenorrhea within the past 1 month;<br>3. Screening for patients who used analgesic medications within 24 hours prior to the screening day;<br>4. Abnormalities such as ulceration or infection of the irradiated skin area;<br>5. Patients with severe psychiatric disorders or other conditions who are unable to accurately perform VAS pain scoring or objectively describe symptoms;<br>6. History of phototherapy allergy, malignant tumor, systemic lupus erythematosus, or bleeding/hemolytic diseases;<br>7. Women who are pregnant, breastfeeding, or expect to become pregnant within 3 months after enrollment;<br>8. Exclude participants who have participated in other medical device or drug clinical trials within the past 1 month on screening day;<br>9. Other circumstances deemed unsuitable for enrollment by the investigator, such as anticipated poor compliance. |
| **Study duration**<br>Clinical trials shall be completed within 12 months after obtaining ethical approval, completing provincial bureau registration, and product availability. |

# I. Sponsor Information

### (I) Sponsor Name

Beijing Charmwin Light Medical Technology Co., Ltd.

### (II) Sponsor's Address

East side of the 1st floor, Building 1, No.3 Yuncheng Street, Beijing Economic-Technological Development Area, Beijing.

### (III) Contact Information of the Sponsor

Jun He (13370082505).

### (IV) Applicant's relevant qualification documents

Relevant qualification documents of the sponsor are attached separately.

### (V) Name, address, contact information, and relevant qualification documents of the agent

Not applicable.

# II. List of All Clinical Trial Institutions and Investigators in the Multicenter Clinical Trial

The list of clinical trial institutions and investigators is provided in the Summary Report of Clinical Trials.

# III. Objectives and Content of Clinical Trials

### (I) Objective

The clinical use of LED red light therapy devices produced by Beijing Charmwin Light Medical Technology Co., Ltd. was evaluated to assess the safety and efficacy of LED red light therapy devices for alleviating primary dysmenorrhea.

### (II) Content

This trial adopted an open, single-group target-value design. Subjects signed informed consent forms and were enrolled only after meeting the eligibility criteria. The LED red light therapy device manufactured by Beijing Charmwin Light Medical Technology Co., Ltd. was used to alleviate primary dysmenorrhea, thereby evaluating the safety and efficacy of the clinical application of the LED red light therapy device produced by Beijing Charmwin Light Medical Technology Co., Ltd.

# IV. Background Information on Clinical Trials

Primary dysmenorrhea refers to a condition where there is no obvious lesion in the reproductive organs and no clear cause, and the symptoms are manifested as lower abdominal pain and distension during menstruation, accompanied by other discomforts such as lumbago,

nausea, vomiting and headache[1]. It is a common complaint among adolescent females, distinguished by menstrual pain and idiopathic pelvic pain. This condition affects approximately 25% of women during their menstrual periods, with an estimated prevalence ranging from 20% to 90%, peaking during adolescence[2]. About 15% of adolescent females report experiencing severe primary dysmenorrhea that prevents them from participating in normal social activities for several days during each menstrual cycle.

The pathogenesis of primary dysmenorrhea is complex. The cessation of progesterone secretion before menstruation, along with uterine and vascular contraction, triggers intense inflammatory responses, leading to uterine ischemia and pain, which constitutes the primary etiology.

In the treatment of primary dysmenorrhea, nonsteroidal anti-inflammatory drugs (NSAIDs) such as ibuprofen, naproxen sodium, and mefenamic acid are the most commonly used medications for alleviating menstrual pain and demonstrate significant efficacy in managing primary dysmenorrhea. It has been reported that oral contraceptives are also effective in relieving menstrual pain by reducing the release of prostaglandins, vasopressin, and oxytocin during menstruation. However, due to their pronounced effects on metabolic processes, these contraceptives are associated with a higher incidence of adverse reactions[3]. For patients who are concerned about adverse effects and prefer not to use NSAIDs or hormonal therapy, non-pharmacological analgesic treatments such as transcutaneous electrical nerve stimulation, acupuncture, far-infrared therapy, local hyperthermia, and low-level laser therapy may be considered. Nevertheless, there is scant evidence to support any particular treatment modality as the first-line option among these options[2].

The LED red light therapy device (experimental instrument) manufactured by Beijing Charmwin Light Medical Technology Co., Ltd. consists of a treatment head, magnetic input base, adjustable abdominal strap, control output connection cable, controller, and power adapter. It utilizes high-purity, high-power narrow-band red light to alleviate primary dysmenorrhea. The primary mechanism of action involves increasing the concentrations of cyclic adenosine monophosphate (cAMP) and nitric oxide (NO) in patients through light exposure, relaxing uterine smooth muscles, enhancing blood flow, reducing inflammation, and restoring normal rhythmic contraction and relaxation of smooth muscles, thereby mitigating dysmenorrhea.

The trial device has passed the registration testing conducted by the Beijing Medical Device Testing Institute, with all parameters complying with national standards and product technical requirements. To evaluate the safety and efficacy of the trial device in alleviating primary dysmenorrhea, this clinical trial protocol is formulated in accordance with the Good Clinical Practice for Medical Devices (GCP), relevant national regulations and ethical requirements, as well as the characteristics and mechanisms of the product.

**Background information references:**

[1] Zhang J, Bai JG, Bai Lixia, et al. Efficacy and safety study of specific wavelength low-power visible light therapy for primary dysmenorrhea [J]. Journal of Practical Gynecological Endocrinology (Electronic Edition), 2018,5(07):3-7.

[2] Hong G Y , Shin B C , Park S N , [2] Hong G Y , Shin B C , Park S N , et al. Randomized controlled trial of the efficacy and safety of self-adhesive low-level light therapy in women with primary dysmenorrhea[J]. International Journal of Gynaecology & Obstetrics the Official Organ of the International Federation of Gynaecology & Obstetrics, 2016, 133(1):37.

[3] Ji Bo, Ren Xiaoxuan, Zhao Yafang, et al. Review on the Pathogenesis and Prevention of Primary Dysmenorrhea [J]. China Modern Medicine Journal, 2008,18(13):4.

## V. Product Features, Structural Composition, Working Principle, Mechanism of Action, and Test Scope

### (I) Product Features

The LED red light therapy device is a home-use therapeutic apparatus that employs high-purity, high-power narrow-band red light to alleviate dysmenorrhea through phototherapy.

### (II) Product Structure Composition, Working Principle, and Mechanism of Action

#### 1. Structural composition

It consists of a treatment head, input magnetic suction base, adjustable abdominal belt, control output connection line, controller, and power adapter.

#### 2. Working Principle and Mechanism of Action

For a long time, low-intensity light therapy (LLLT) has been primarily used to alleviate muscle pain in patients. LLLT can induce bioactivity: stimulating ATP synthesis, cellular metabolism, protein synthesis, and cell proliferation; inducing anti-inflammatory responses; enhancing neural function; and producing physiologically active substances such as nitric oxide (NO), cyclic adenosine monophosphate (cAMP), and endorphins.

In mammalian cells, certain colors of light increase intracellular cAMP concentrations. The elevated intracellular cAMP levels lead to relaxation of uterine smooth muscle. Additionally, low-intensity light can enhance NO production, a well-known vasodilator and neurotransmitter that increases blood flow and reduces inflammation. Consequently, the relaxation of uterine smooth muscle may increase blood flow to the smooth muscle tissue. Some damaged smooth muscles can obtain oxygen and nutrients by enhancing blood supply, thereby restoring normal rhythmic contraction and relaxation of the smooth muscle, which alleviates dysmenorrhea.

### (III) Scope of the trial

Participants diagnosed with primary dysmenorrhea.

## VI. Scope of Product Indications, Contraindications, and Precautions

### (I) Scope of Application

Primary dysmenorrhea.

### (II) Contraindications

1. Photosensitivity, systemic lupus erythematosus;
2. Unstable blood pressure, arrhythmia, and pacemaker implantation;

3. Tumor patients;

4. Patients with bleeding disorders or hemolytic diseases;

5. Patients with acute suppurative inflammation of the superficial abdominal layer;

6. Individuals with pregnancy planning intentions;

7. Other patients unsuitable for irradiation.

**(III) Precautions**

1. The replacement of the therapeutic controller for this product should be performed under the guidance of the manufacturer.

2. Individuals without professional maintenance experience from the manufacturer are prohibited from disassembling this product.

3. For maintenance or shipment of all instrument components, please contact the manufacturer.

4. During use, increased menstrual flow and early or delayed menstruation by 5-7 days may occur, which are all normal phenomena. If symptoms such as dry skin, itching, or flushing appear, self-administration of moisturizing treatments may be considered when necessary.

## VII. Overall Design

### (I) Experimental Design

#### 1. Objective of the trial

The objective of this trial was to evaluate the safety and efficacy of LED red light therapy device for alleviating primary dysmenorrhea.

**(1) Main objectives**

Evaluate whether the primary clinical performance (response rate) of the LED red light therapy device meets the predefined target values.

**(2) Secondary objectives**

Evaluate the secondary clinical performance (COX Dysmenorrhea Symptom Scale scores, participant experience assessment, VAS scores) and safety (life signs, adverse events) of the LED red light therapy device, as well as device defects.

#### 2. Selection of Test Methods and Rationale

The single-group target value method was selected for this trial for the following reasons:

1) No similar products are currently available domestically, making positive parallel control unfeasible. 2) Current clinical practice for primary dysmenorrhea predominantly employs psychological interventions, pharmacotherapy, or surgical procedures as standard treatments. These approaches exhibit significant differences in therapeutic principles and expected outcomes (e.g., ease of use, non-invasiveness, painlessness, and absence of medication) compared to the experimental device's design objectives. The achievable therapeutic efficacy also varies substantially, with no conclusive evidence indicating superiority among these methods. Therefore, standard treatment modalities are excluded from control group consideration.

Therefore, this trial adopted a single-group target value approach. By synthesizing literature on phototherapy for dysmenorrhea relief (see sample size calculation basis), the achievable therapeutic efficacy from the literature was established as the target value for this trial.

**3. Measures to reduce and avoid bias**

This trial adopted a single-group target value approach, with the observed bias being non -synchronous control bias. Strict inclusion/exclusion criteria for subjects, commonly used clinical efficacy evaluation methods, and standardized assessment time points were established to control selection bias and evaluation bias.

**4. Experimental medical devices**

Table 1 Basic Information of Test Devices

| product name | model | production unit |
|---|---|---|
| LED Red Light Therapy Device | CY-1002C | Beijing Charmwin Light Medical Technology Co., Ltd. |

**5. Subject Selection**

**(1) Eligibility criteria**

1) Female individuals aged 13–35 years (inclusive of 13 and 35 years old);

2) Diagnosed with primary dysmenorrhea;

3) Regular menstrual cycle: 28±7 days;

4) Individuals who self-reported a mean VAS pain score ≥4 points during the last two menstrual cycles;

5) Voluntary participation in the trial, with informed consent signed by the subject and/or one guardian.

**(2) Exclusion criteria**

1) Secondary dysmenorrhea confirmed by gynecological examination and abdominal ultrasound due to gynecological diseases such as endometriosis, pelvic inflammatory disease, adenomyosis, or other conditions;

2) Screening for oral contraceptives and/or central nervous system-acting medications and/or prior receipt of physical therapy related to dysmenorrhea within 1 month prior to the study day;

3) Screening for analgesic medication use within 24 hours prior to the day of screening;

4) Abnormalities such as ulceration or infection of the irradiated skin area;

5) Patients with severe psychiatric disorders or other conditions who are unable to accurately perform VAS pain scoring or objectively describe symptoms;

6) History of phototherapy allergy, malignant tumor, systemic lupus erythematosus, or bleeding/hemolytic diseases;

7) Women who are pregnant, breastfeeding, or expect to become pregnant within 3 months after enrollment;

8) Exclude participants who have participated in other medical device or drug clinical trials within the past 1 month on screening day;

9) Other circumstances deemed unsuitable for enrollment by the investigator, such as anticipated poor compliance.

**(3) Exclusion criteria**

1) Violation of critical enrollment criteria;

2) Un-treated individuals;

3) No records were obtained during the trial process;

4) Concurrent use of other devices that may affect efficacy assessment;

**(4) Criteria for stopping the trial**

The conditions for trial discontinuation include subject withdrawal and trial termination.

**Exit trial**

[1] Withdrawal of informed consent by the subject; [2] Serious violation of the clinical trial protocol; [3] The investigator deems the subject no longer suitable for continued participation in the clinical trial; [4] Pregnancy during the clinical trial period; [5] Subject death; [6] Subject loss to follow-up; [7] Sponsor request to terminate the clinical trial; [8] Other reasons for withdrawal.

**Withdrawal of subjects from the study**

[1] All participants who withdraw from the study must retain complete source data and original documentation. After withdrawal, investigators should maintain contact through

multiple channels (e.g., phone calls, emails) to understand the reasons for participation. [2] The termination date and rationale for clinical trials must be meticulously documented in case reports. [3] For participants who discontinued due to adverse events, if a causal relationship with the investigational device is confirmed, this must be recorded in case reports and reported to the sponsor. Follow-up must continue until the adverse event resolves or stabilizes.

**Trial termination**

It refers to the situation where a clinical trial is halted halfway before it has been completed as per the protocol. The main purpose is to protect the rights and interests of the subjects and ensure the quality of the trial. Except for the following [5], the decision shall be made through mutual consultation between the sponsor and the researcher: [1] occurrence of severe safety issues during the trial; [2] demonstration of suboptimal efficacy or clinical ineffectiveness of the device, rendering it clinically irrelevant; [3] identification of critical protocol violations that impede device effectiveness evaluation; [4] sponsor-initiated request for termination; [5] The National Medical Products Administration ordered the termination of clinical trials for certain reasons.

**(5) Enrollment time**

After signing the Informed Consent Form, participants are required to complete baseline assessment. Investigators will screen and evaluate whether they meet the inclusion/exclusion criteria. If the participant qualifies for enrollment, the corresponding treatment protocol will be administered. The trial is expected to initiate participant screening and enrollment in March 2022.

**(6) Expected overall duration of clinical trials and rationale for its determination**

The anticipated total duration of the clinical trial includes pre-trial preparation, case enrollment, investigator review time, subject treatment duration, subject follow-up period, data analysis, and summary report preparation. Due to factors such as hospital outpatient/hospitalization volume and subject compliance, the estimated clinical study cycle (covering full case enrollment, investigator review time, subject treatment duration, and subject follow-up period) is 9 months, with an overall duration of 12 months.

**(7) Expected duration of participation for each subject**

This trial includes a screening phase and a follow-up phase, with an estimated participant duration of 3 months.

**(8) Number of subjects required for clinical trials**

The total sample size was 120 cases.

**6. Methods for efficacy evaluation**

**(1) Explanation of efficacy parameters**

**Main evaluation indicators:**

Response rate: The efficacy was assessed based on visual analogue scale (VAS) scores, with the therapeutic effect evaluated during the third menstrual cycle after enrollment serving as the primary endpoint.

**Secondary evaluation indicators**

COX Dysmenorrhea Symptom Scale Score: This scale is a professional tool for evaluating dysmenorrhea and can be used to assess the efficacy of treatment for primary dysmenorrhea.

Subject experience evaluation: The test equipment is for home use. Whether the subjects can have a good experience when using it independently is a key aspect that this test focuses on.

VAS scores (during subject screening period, and during the first, second, and third menstrual cycles after enrollment): VAS scores represent the most widely used method for pain intensity assessment.

Response rate: The efficacy evaluated during the first and second menstrual cycles after enrollment was used as a secondary evaluation indicator.

**(2) Methods and timing selection for evaluating, recording, and analyzing efficacy parameters**

1.Efficieny evaluation [1]:

Markedly effective: VAS score reduction rate ≥70%;

Effective: VAS score reduction rate of 30%-69%;

Ineffective: VAS score reduction rate ≤29%.

The rate of VAS score reduction = ((VAS score at screening stage-VAS score after treatment) / VAS score at screening stage) × 100%.

Effective = Markedly effective + Effective.

Records: Record VAS scores and calculate the VAS score decline rate. Determine the corresponding therapeutic efficacy based on the evaluation criteria. The efficacy assessed during the first and second menstrual cycles after enrollment serves as secondary evaluation indicators, while the efficacy evaluated during the third menstrual cycle after enrollment serves as the primary evaluation indicator.

Analysis: According to the statistical analysis plan.

Time: Screening period, first menstrual cycle after enrollment, second menstrual cycle, and third menstrual cycle.

**2. COX Dysmenorrhea Symptom Scale Score [2]**

Evaluation: Subjects were assessed using the COX Dysmenorrhea Symptom Scale and their scores were statistically analyzed.

Record: Document the scores on the COX Dysmenorrhea Symptom Scale, with separate scoring for symptom severity and duration.

Analysis: According to the statistical analysis plan.

Time: Screening period, first menstrual cycle after enrollment, second menstrual cycle, and third menstrual cycle.

**3. Subject User Experience Evaluation**

Evaluation: calculated using a 5-order classification method

1) This is extremely inconvenient and unsatisfactory.

2) Inconvenience, dissatisfaction,

3) Generally portable, generally satisfactory,

4) Portable and user-friendly,

5) Highly portable and very satisfactory.

Record: Grading of evaluation records. Items 1)-3) are defined as poor user experience, while items 4)-5) are defined as good user experience.

Analysis: According to the statistical analysis plan.

Time: During the first, second, and third menstrual cycles after enrollment.

**4. VAS score**

Evaluation: Provide a 10 cm ruler on paper with the left end of the horizontal line marked as 0 (indicating no pain) and the right end marked as 10 (indicating severe pain), while the middle section represents varying degrees of pain. Instruct the subject to mark a point on the horizontal line according to their subjective perception to indicate the intensity of pain.

Record: Investigators recorded VAS scores based on designated markers.

Analysis: According to the statistical analysis plan.

Time: Screening period, first menstrual cycle after enrollment, second menstrual cycle, and third menstrual cycle.

**Reference literature for efficacy evaluation indicators:**

[1] Xuelian Fan, Ping Xue, Liangping Wang. Efficacy observation of super laser pain therapy device combined with traditional Chinese medicine acupoint therapy for dysmenorrhea [J]. World Latest Medical Information Digest, 2015,000(031):84-85.

[2] Yuxia Ma, Haiyang Ma, Shaozong Chen, et al. Validation of the Chinese Version of the COX Dysmenorrhea Symptom Scale: Reliability and Validity [J]. Shandong Journal of China Medical University, 2015,39(01):5-7.

**7. Safety Evaluation Methods**

(1) Description of safety parameters

Life signs: respiration, pulse, blood pressure, body temperature.

Adverse event: refers to an unfavorable medical event occurring during clinical trials, regardless of whether it is device-related.

Device defects: Refers to unreasonable risks that may endanger human health and life safety during clinical trials when medical devices are used under normal conditions, such as labeling errors, quality issues, malfunctions, etc.

(2) Methods and timing selection for evaluating, recording, and analyzing safety parameters

**1. Vital signs**

Evaluation: Assess the vital signs of the subject at the corresponding time points.

Record: Record respiration, pulse, blood pressure, and body temperature.

Analysis: According to the statistical analysis plan.

Time: Screening period, first menstrual cycle after enrollment, second menstrual cycle, and third menstrual cycle.

**2. Adverse Events**

Evaluation: Monitor for the occurrence of adverse events.

Record: Document the category, severity, and causality of adverse events.

Analysis: According to the statistical analysis plan.

Time: From the time of using the device until the third menstrual period.

**3. Mechanical defects**

Evaluation: Observe for the occurrence of device defects.

Record: Document the category of device defects.

Analysis: According to the statistical analysis plan.

Time: From the time of using the device until the third menstrual period.

**(II) Trial Procedure**

**1. Experimental procedure**

| **Stage** | **Screening period** | **Follow-up period** | | |
|---|---|---|---|---|
| | **Visit 1** | **Visit 2** | **Visit 3** | **Visit 4** |
| **Observation time point** | **-14 to 0 days** | **First menstrual period after enrollment** | **The second menstrual period after enrollment** | **The third menstrual period after enrollment** |
| Informed consent | X | | | |
| Inclusion/Exclusion Criteria | X | | | |
| Baseline data [a] | X | | | |
| Concomitant medication [b] | X | X | X | X |
| Previous medical history and comorbid conditions [c] | X | | | |
| Uterine pregnancy | X | | | |
| Vital sign [d] | X | X | X | X |
| Abdominal ultrasound and gynecological examination | X | | | |
| VAS grade | X | X | X | X |
| COX Dysmenorrhea Symptom Scale Score | X | X | X | X |
| Subject User Experience Evaluation | | X | X | X |
| Device defect | | X | X | X |

| Adverse event [e] | | X | X | X |
|---|---|---|---|---|

**Observation items and indicators:**

a) Baseline data included demographic information (age, height, weight, date of birth) and self-reported mean VAS scores for the last 2 menstrual cycles;

b) Record medications used for the treatment of primary dysmenorrhea, such as nonsteroidal anti-inflammatory drugs (NSAIDs), oral contraceptives, and calcium channel blockers;

c) Pre-existing medical history screening including: treatment history of menstrual pain within the previous 1 year, history of phototherapy allergy, history of malignant tumors, history of systemic lupus erythematosus (SLE), and history of bleeding/hemolytic diseases;

d) Vital signs: respiration, pulse, blood pressure, body temperature;

e) Record adverse events occurring after device use. The following scenarios may exempt reporting of serious adverse events: ① Hospitalization for the underlying disease itself, not due to new adverse reactions or exacerbation of pre-existing conditions: e.g., chemotherapy for tumors; ② Administrative admissions, such as annual health check-ups; ③ Voluntary planned admissions unrelated to clinical response events, such as cosmetic surgery; ④ Scheduled elective hospital admissions for pre-trial planned surgeries; ⑤ Non-hospitalization care procedures completed on the same day without involving hospitalization protocols; ⑥ Short-term stays in observation rooms; ⑦ Other settings, including rehabilitation centers, nursing centers, and home-based professional care.

**Note :**

1) Screening period (-14 to 0 days): During this phase, candidates undergo screening. Those who pass the screening will receive VAS scoring and COX Dysmenorrhea Symptom Scale scoring during the premenstrual period (before treatment initiation).

2) Visit 2-4: Duration from enrollment to the third menstrual period of the participant. Instruct patients to use the device 7 days before each of the first, second, and third menstrual periods after enrollment (30 minutes per session, once daily), discontinue use during menstruation, and undergo follow-up during the menstrual cycle.

3) During the trial period, non-steroidal anti-inflammatory drugs (NSAIDs), oral contraceptives, calcium channel blockers, and other primary dysmenorrhea treatment medications such as analgesics, central nervous system drugs, as well as related physical therapies for dysmenorrhea are generally prohibited. From an ethical perspective, the use of analgesics is permitted when subjects experience exacerbated pain or difficulty in pain control, but researchers must document such cases.

**2. Standardized Use of Medical Devices**

For usage instructions, please refer to the package insert.

**(III) Monitoring Plan**

**1. Requirements for monitors**

The monitor shall adhere to the Standard Operating Procedures for Monitoring Clinical Trials of Investigational Medical Devices and ensure that clinical trials are conducted in accordance with the protocol.

**2. Schedule**

The frequency of monitoring depends on the enrollment progress and should be conducted at least once per month at the clinical trial site. Adjustments may be made based on actual clinical circumstances.

**3. Monitoring Implementation**

(1) Prior to the trial, confirm that the clinical trial site has adequate conditions, including personnel allocation and training meeting requirements, complete laboratory equipment with proper operational status, anticipated sufficient number of participants, and trial participants familiar with trial requirements.

(2) Monitor whether clinical trial institutions and investigators comply with relevant regulations, standards, and clinical trial protocols during the pre-, mid-, and late phases of the trial.

(3) Confirm that each subject has signed an informed consent form prior to participating in the clinical trial, and understand the subject's eligibility criteria as well as the progress of the trial. Clearly and truthfully document any follow-up visits that the investigator failed to conduct, trials that were not performed, examinations that were not conducted, and whether corrections were made for errors or omissions. For revised informed consent forms, confirm that affected subjects who have not completed the clinical trial process have re-signed them.

(4) Verify that all case report forms are correctly filled out and consistent with the original data; all errors or omissions have been corrected or noted, signed by the investigator and dated; for each trial, the disease type, total number of cases, as well as case characteristics such as gender, age, and treatment efficacy should be confirmed and recorded.

(5) Confirm that records of subjects withdrawing from clinical trials or failing to comply with the requirements specified in the informed consent form are maintained, and discuss such cases with the investigator.

(6) Confirm that all adverse events, complications, and other device defects are documented, with severe adverse events and device defects that may lead to severe adverse events being reported and recorded within the specified timeframe.

(7) Monitor the supply, use, maintenance, transportation, receipt, storage, distribution, disposal, and recovery of investigational medical device samples.

(8) Supervise the regular maintenance and calibration of relevant equipment during clinical trials.

(9) Ensure that all clinical trial-related documents received by the investigator are in the latest version.

(10) A written report shall be prepared after each monitoring session, which shall include the name of the monitor, monitoring date, monitoring time, monitoring location, monitoring content, name of the investigator, project completion status, identified issues, conclusions, and corrective actions taken for errors or omissions.

## VIII. Statistical Considerations

### (1) Statistical design, methods, and analytical protocols

#### 1. General Principles

Statistical analysis will be performed using SAS 9.4 (or higher version) statistical analysis software.

The statistical analysis of this trial primarily involved descriptive analysis. Quantitative indicators were described by calculating mean, standard deviation, median, minimum value, maximum value, lower quartile (Q1), and upper quartile (Q3). Categorical indicators were described by presenting the number of cases and percentages for each category.

#### 2. Statistical Analysis Plan

#### 1) Selection and Completion Status

List the enrollment and completion rates of cases at each center along with the reasons for dropout, and describe the number of enrolled cases and the dataset for safety and efficacy analysis. Provide a list of cases that did not enter the PPS.

#### 2) Baseline analysis

Descriptive analysis: Categorical data were described using frequency and composition ratio, while continuous data were described using mean, standard deviation, maximum value, minimum value, median, and 95% confidence interval.

Descriptive analysis methods were used for baseline demographic data analysis.

**3) Validity analysis**

**Main Evaluation Indicators**

The test hypothesis is as follows:

$H_0$ : $P_T \leq P_0$

$H_1$ : $P_T > P_0$

α=0.025 (one-sided).

Among them, $P_T$ is the overall parameter of the primary evaluation index; $P_0$ is the target value of the primary evaluation index.

The datasets selected for the primary evaluation indicators were the FAS and PPS sets.

**Secondary indicators**

The datasets selected for secondary indicators were the FAS set and PPS set, with statistical analysis conducted in accordance with the aforementioned general principles.

**4) Safety Analysis (Safety analysis based on SS set)**

① Vital sign

The list provides detailed descriptions of vital signs, listing abnormal items and their respective percentages.

② Adverse event

The types, severity, and relationship to the investigational device of all adverse events during the trial period will be described in a tabular format.

Adverse events will be listed under the categories of all adverse events and related adverse events. For conservative purposes, related adverse events will include those that are "definitely associated," "likely associated," or "possibly associated" with the investigational device.

③ Mechanical defect

All device defects during the trial period will be described in a list.

**(II) Calculation of sample size**

**1. Total sample size**

Sample size calculation formula: $n_T = \frac{[Z_{1-\alpha}\sqrt{P_0(1-P_0)} + Z_{1-\beta}\sqrt{P_T(1-P_T)}]^2}{(P_T-P_0)^2}$

Parameter settings: 1. α, significance level: In this trial, α=0.025 (one-sided) was adopted;

2. 1-β, confidence level: In this trial, β=0.20;

3. $P_0$ and $P_T$, the target values of primary evaluation indicators for the test product ($P_0$) and the rate of primary evaluation indicators for the test product ($P_T$): This trial

primarily evaluates efficacy rate as the primary indicator, with an assumed efficacy rate ($P_T$) of 90%. The target efficacy rate ($P_0$) for this trial is set at 80%.

Through calculation, the sample size of 107 cases, 10% consider the fall off and clinical operation, the required sample size of 120 cases.

**Rationale for target value establishment:**

The target value was established based on the efficacy of light therapy in alleviating primary dysmenorrhea reported in Chinese and English literature.

1. Literature [1] reported that low-power visible light at specific wavelengths demonstrated an efficacy rate of 76.3% in alleviating primary dysmenorrhea (the criterion for efficacy evaluation: a reduction of more than 10% in VAS scores from baseline was considered effective).
2. Literature [2] reported that compared with baseline VAS scores, the change in VAS score at the study endpoint was-4.34±2.22 points in the trial group (self-adhesive low-power light). This literature only reported VAS scores and did not report efficacy rates.
3. Literature [3-4] reports that a reduction of 0.9-1.5 points on the 10-point VAS scale can be considered effective.

Through literature review, reference [1] was identified as having significant reference value. The standard for therapeutic efficacy in this trial was defined as The rate of VAS reduction was ≥30%, exceeding the standard in literature [1] (a reduction greater than 10%). The efficacy rate reported in literature [1] was 76.3%, while the target value of 80% in this trial surpassed the efficacy rate achieved in literature [1]. It can be concluded that both the efficacy evaluation criteria and the expected target of this trial were higher than those in literature [1].

Literature [3-4] indicates that an absolute change in VAS score of 0.9-1.5 is considered effective. In this trial, the VAS score reduction rate was calculated as ((VAS score at screening stage-VAS score after treatment) / VAS score at screening stage) × 100%. Assuming that the pre-treatment VAS score of enrolled subjects was 4 points, according to the evaluation criteria (a VAS reduction rate ≥30% is deemed effective), a minimum reduction of 1.2 points was required to be evaluated as effective. The evaluation criteria for this trial are listed as follows:

| Baseline VAS score | Computational formula | Lowest effective standard | Achieve the minimum effective standard |
|---|---|---|---|

| | | | |
|---|---|---|---|
| 4 | Rate of decline in VAS score= (Screening period VAS score-VAS score after therapy)/Screening period VAS score × 100% | A reduction rate of ≥ 30% in VAS is considered effective. | A decrease of at least 1.2 is required to be considered effective. |
| 5 | | | A decrease of at least 1.5 is required to be considered effective. |
| 6 | | | A decrease of at least 1.8 is required to be considered effective. |
| 7 | | | A decrease of at least 2.1 is required to be considered effective. |
| 8 | | | A decrease of at least 2.4 is required to be considered effective. |

It can be observed that in this trial, the criteria for efficacy evaluation became stricter as the baseline VAS scores increased compared to those defined in the literature [3-4].

Therefore, this trial comprehensively considered the literature data and incorporated the opinions of clinical experts, setting the target value at 80%.

**Reference for target value establishment:**


[1] Zhang J, Bai JG, Bai Lixia, et al. Efficacy and safety study of specific wavelength low-power visible light therapy for primary dysmenorrhea [J]. Journal of Practical Gynecological Endocrinology (Electronic Edition), 2018,5(07):3-7.

[2] Hong G Y , Shin B C , Park S N , [2] Hong G Y , Shin B C , Park S N , et al. Randomized controlled trial of the efficacy and safety of self-adhesive low-level light therapy in women with primary dysmenorrhea[J]. International Journal of Gynaecology & Obstetrics the Official Organ of the International Federation of Gynaecology & Obstetrics, 2016, 133(1):37.

[3] Kelly AM (1998) Does the clinically significant difference in visual analogue scale pain scores vary with gender, age, or cause ofpain? Acad Emerg Med 5(11):1086-1090

[4] Abbott J, Hawe J, Srivastava P, Hunter D, Garry R (2001) Intraperitoneal gas drain to reduce pain after laparoscopy: randomized masked trial. Obstet Gynecol 98(1):97-100


**2. Number of clinical trial cases per disease category and rationale for determination**

The trial enrolled subjects with primary dysmenorrhea, indicating that the current study focuses solely on a single indication without further classification.

**3. Minimum and maximum number of subjects per clinical trial site and rationale in multicenter clinical trials**

This trial will be conducted simultaneously at two clinical trial sites. In principle, the enrollment numbers across centers will be allocated as evenly as possible (sample size 1:1

distribution) to ensure adequate center representativeness. However, considering feasibility and enrollment progress, the enrollment numbers may be adjusted based on actual circumstances (e.g., adopting competitive enrollment methods) to maintain relatively balanced enrollment scales across each center.

**(III) Significance level and confidence level of clinical trials**

1. Primary evaluation indicators, with a significance level of $\alpha = 0.025$ (one-sided) and a power of $1\text{-}\beta = 0.8$.

2. Other hypothesis tests, with a significance level of $\alpha=0.05$ (two-tailed) and a power of $1\text{-}\beta=0.8$.

**(IV) Expected dropout rate**

The expected dropout rate shall not exceed 10%.

**(V) Qualification/Nonqualification Criteria for Clinical Trial Results**

Perform hypothesis testing on the primary evaluation indicators. If P ≤ " is met, reject $H_0$ and conclude that the trial product meets the design requirements. Alternatively, if the lower limit of the 95% confidence interval for the primary evaluation indicator of the test product exceeds the target value, it is concluded that the test product meets the design requirements and the clinical trial results are qualified; otherwise, the clinical trial results are unqualified.

**(VI) Criteria and rationale for terminating trials based on statistical reasons**

This trial does not include interim analysis and will not be terminated based on statistical reasons.

**(VII) Statistical methods for all data, including missing, unused, or erroneous data (including those withdrawn or discontinued during the study) and methods for handling unreasonable data**

Prior to trial initiation, a statistical analysis plan shall be formulated. After database locking, statistical analysis programming and logic validation programming shall be conducted in accordance with the statistical analysis plan. Subsequently, simulated database testing shall be performed, and a statistical analysis report shall be issued based on the results.

1. Missing value handling: For missing data, processing will be performed according to the dataset partitioning. For primary evaluation data, the LOCF method will be employed for imputation, while missing values in secondary evaluation indicators will not be processed.

2. Unreasonable data processing: During data management, logical verification of data in the database will be conducted. If unreasonable data is identified, a query form will be issued to researchers to raise questions. Adjustments to the unreasonable data will be made based on the researchers' written responses until all unreasonable data is resolved, after which the database can be locked.

3. Error data handling: During data management, quality control will be performed on data in the database. Identified erroneous data will be raised to researchers via query forms, and corrections will be made based on their written responses until all errors are rectified before the database can be locked.

**(VIII) Procedures for reporting deviations from the original statistical plan**

In principle, the aforementioned general principles of statistical analysis, analytical content and methods, data processing principles, and other relevant statistical analysis plans shall not undergo significant modifications. If changes are required, they must be determined through joint discussion among the biostatistician, investigators, ethics committee, and sponsor, and shall be documented with justification in the revised clinical trial protocol and clinical trial summary report.

**(IX) Selection criteria and rationale for subjects included in the analysis**

Full Analysis Set (FAS): To approximate the analysis set for all subjects as closely as possible, it should include all enrolled subjects who used the device at least once.

Per Protocol Set (PPS): A subset of the full analysis set that includes treatments specified in the accepted protocol, observational data available for primary endpoints, and subjects who did not significantly deviate from the trial protocol.

Safety Analysis Set (SS): Includes all enrolled subjects who used the device once and underwent safety evaluation.

**(X) Exclusion of special information and rationale when verifying hypotheses (if applicable)**

N/A

## IX. Data Management

The data management for this clinical trial is the responsibility of the data management party. During the trial process, it is essential to ensure the authenticity, completeness, and confidentiality of clinical trial data. The data management process must comply with the regulations for medical device clinical trial management to guarantee the traceability of clinical trial data.

(1) Database establishment and management

Based on the specific content of the case report form, data managers utilize software such as EpiData or EXCEL to encode all study indicators, establish a database, and perform data entry, verification, and export. Dedicated personnel are assigned to manage the database.

(2) Monitoring of raw data

The monitor oversees whether the trial is conducted in accordance with the protocol, verifies that all case report forms are correctly and completely filled out, and ensure full consistency with the original data. Any errors or omissions should be promptly corrected by the investigator. Modifications must retain original records and be clearly visible, with corrections requiring the investigator's signature and date notation. Upon completion of case report forms, the monitor must promptly retrieve the first copy and submit it to the data management department. Investigators, monitors, and data administrators are required to verify the retrieved case report forms.

(3) Data Entry and Modification

Data entry is performed by the data administrator.

For questions raised in case report forms, the data administrator will issue a Data Query and submit it to the investigator through the monitor. The investigator must respond promptly and provide feedback. Based on the investigator's responses, the data administrator will confirm and modify the data, and may issue additional Data Queries if necessary.

(4) Data Verification

Data verification includes manual review, computerized program verification, and data verification meetings. For data issues identified during verification, such as missing values, anomalies, or logical errors, the data management department shall promptly issue a data verification report, which must be submitted to the investigator for verification and confirmation before any modifications are made.

(5) Database locking

Upon completion of clinical trial data entry, the sponsor, investigators, and data management team jointly verify the data. After confirming the accuracy of the data, the database is locked. The locked data is then exported for statistical analysis.

(6) Data archiving

Upon completion of the clinical trial, the data management department shall transfer the complete database and relevant documents to the sponsor.

The sponsor shall retain the device until its discontinuation of use.

(The above describes the operational procedures for paper-based Case Report Forms (CRFs). If E-CRF is used in clinical trials, the corresponding data management procedures for E-CRF may be followed.)

# X. Feasibility Analysis

### (I) Probability Analysis of Success

1. This trial was designed and implemented in compliance with relevant regulations/norms such as the Good Clinical Practice for Medical Devices (GCP) and the Guiding Principles for the Design of Clinical Trials of Medical Devices. The scientific validity of the clinical trial protocol was ensured, and the trial also met regulatory requirements.

2. The raw materials for the experimental device comply with national standards, and their safety has been recognized. The device has passed registration inspection and a qualification report has been issued. Strict quality control measures are implemented prior to factory release, ensuring no defective products are produced.

3. The indicators used in this experimental process are easy to observe and record.

### (II) Analysis of Failure Probability

The primary causes of treatment failure or suboptimal efficacy may be associated with the following factors:

1. Injury to the subject caused by the device itself;
2. The product exhibits extremely rare manufacturing defects;
3. Poor subject compliance prevents the acquisition of valid trial data for statistical analysis;
4. The product manual fails to provide effective guidance to users, resulting in poor operability of the product;

In addition to the aforementioned reasons, this study may also encounter other unpredictable risks that could lead to research failure.

# XI. Quality Control in Clinical Trials

(1) Standard Operating Procedure (SOP) Training

Establish standardized SOPs and provide training for all investigators. Investigators should be proficient in the specific implementation details and operational procedures of the clinical trial protocol, as well as standardized recording methods and evaluation criteria.

During the trial process, investigators must strictly adhere to the clinical protocol and exercise rigorous control over all factors and indicators that significantly impact trial outcomes (including inclusion criteria, exclusion criteria, efficacy evaluation indicators, safety evaluation indicators, etc.). When completing case report forms, all content must be recorded accurately, comprehensively, and meticulously in accordance with the specified requirements to ensure the completeness, authenticity, and reliability of the case report form data.

Establish standard operating procedures (SOPs) related to clinical trial quality control for compliance by clinical trial institutions and investigators.

(II) Clinical Monitoring

For details, please refer to the implementation of supervision and inspection.

## XII. Ethical Issues in Clinical Trials and Informed Consent

(I) Ethical considerations

Medical device clinical trials shall adhere to the ethical principles established in the Declaration of Helsinki adopted by the World Medical Assembly. Ethics committees shall uphold the principles of ethics and science to review and oversee the implementation of clinical trials.

During the clinical trial process, any revisions to documents such as the clinical trial protocol and informed consent forms, requests for deviations, or resumption of suspended clinical trials must be implemented only after obtaining written approval from the ethics committee.

The ethics committee shall conduct follow-up supervision of clinical trials conducted by the clinical trial institution. In cases where the rights and interests of participants cannot be safeguarded, the committee may at any time issue a written request to suspend or terminate the clinical trial.

Clinical trials that have been suspended shall not be resumed without the consent of the ethics committee.

(II) Approval of the trial protocol

Clinical trials shall obtain approval from the ethics committee of the medical device clinical trial institution. Clinical trials may only be conducted after obtaining approval from the ethics committee.

(III) Informed consent process and informed consent form text

Prior to participant enrollment in a clinical trial, investigators must provide comprehensive explanations regarding the trial details to the subject and/or the guardian of a person without civil capacity or with limited civil capacity, including known and foreseeable risks as well as potential adverse events. Following thorough and detailed disclosure, the subject and/or their guardian must sign the informed consent form with their name and date, and the investigator must also sign the form with their name and date.

The informed consent form shall be prepared in language and text that can be understood by the subject and/or their guardian. The informed consent form shall not contain any content that may cause the subject to waive their legitimate rights and interests or exempt the clinical trial institution, investigators, sponsors, or their agents from their responsibilities.

The informed consent form shall specify the date of formulation or the date of the revised version. If the informed consent form is amended during the trial process, the revised version must obtain additional approval from the ethics committee prior to implementation. After submitting the revised informed consent form to the clinical trial institution, all participants whose trial procedures remain ongoing and who may be affected shall sign the newly revised informed consent form.

The text of the informed consent form is attached separately.

## XIII. Provisions on Reporting Adverse Events and Device Defects

(I) Adverse Events

**definition**

Refers to adverse medical events occurring during clinical trials, regardless of whether they are device-related.

**Severity Assessment Criteria**

(1) Mild: Inquiry revealed;

(2) Moderate: Active narration with tolerance;

(3) Severe: Objective manifestations are present, with intolerable symptoms.

**Criteria for determining causality with medical devices**

(1) Relevance confirmation: The sequence of device use and reaction occurrence is logical; the reaction ceases after discontinuation of the device, or rapidly alleviates or improves; upon re-administration, the reaction recurs and may significantly worsen;

supported by literature evidence; and confounding factors such as the original disease have been excluded.

(2) Highly likely related: No history of reusing medical devices, consistent with "definitely related," or although there may be concurrent use of other devices or medications, the possibility of adverse reactions caused by concurrent use of other devices or medications can be essentially ruled out.

(3) Potential relevance: The use of medical devices is closely associated with the timing of adverse reactions, as evidenced by literature; however, multiple devices or medications may have triggered the adverse reactions, or factors contributing to disease progression cannot be excluded.

(4) Potential irrelevance: The adverse reaction shows no strong correlation with the duration of device use, and its clinical manifestations do not align with known adverse reactions associated with the device. The progression of the underlying disease may also present with similar clinical features.

(5) Irrelevance: The adverse reaction does not match the known types of reactions in the trial; the reaction may be induced by the subject's clinical status or the treatment regimen administered during the trial, may resolve with improvement in the subject's condition or discontinuation of other treatments, or may recur after re-administration of alternative therapeutic approaches.

**Outcome determination**

(1) Death: Results in the termination of life (cause of death and time of death must be collected);

(2) Continued: No improvement/recovery of symptoms after treatment;

(3) Complete resolution without sequelae: After treatment, symptoms are fully resolved without any residual manifestations;

(4) Disappearance with sequelae: After treatment, symptoms have resolved but sequelae remain (the name or manifestations of the sequelae should be noted during documentation);

(5) Unknown: The condition is unclear, with no follow-up observation or recording conducted, or the subject refused participation.

(II) Serious Adverse Event (SAE)

Refers to events occurring during clinical trials that result in: death; severe deterioration of health in patients, users, or others, including: fatal diseases or injuries, permanent defects in body structure or function, hospitalization or prolonged hospital stay,

and the need for medical or surgical interventions to prevent permanent defects in body structure or function; fetal distress, fetal death, or congenital anomalies/congenital defects.

(III) Instrumental Defects

Refers to unreasonable risks that may endanger human health and life safety during clinical trials when medical devices are used under normal conditions, such as labeling errors, quality issues, malfunctions, etc.

If device defects that may lead to serious adverse events are identified, investigators must promptly inform the sponsor. The sponsor shall report to the registered regulatory authority and the corresponding health commission department within 5 working days of notification, while simultaneously notifying other participating clinical trial institutions and investigators. The sponsor's medical device clinical trial management department shall then promptly notify the ethics committee of the respective clinical trial institution.

(IV) Reporting Procedures and Contact Information

1. Reporting Procedures

For all serious adverse events (SAEs) occurring during clinical trials, investigators must immediately administer appropriate treatment measures to participants, submit written reports to the medical device clinical trial management department of the affiliated clinical trial institution, and notify the sponsor in writing. The medical device clinical trial management department shall submit written reports to the relevant ethics committee and provincial regulatory authorities where the clinical trial institution is located within 24 hours. In cases of death events, both the clinical trial institution and investigators must provide the ethics committee and sponsor with all required supplementary documentation.

For Serious Adverse Events (SAEs) and medical device defects that may lead to SAEs, the sponsor shall report to the registered regulatory authority within 5 working days, while simultaneously notifying other clinical trial institutions and investigators participating in the trial. The medical device clinical trial management department of the participating clinical trial institution shall promptly inform the ethics committee of that institution.

The case report form of SAE should record event-related information as completely and detailedly as possible. For fatal events, the clinical trial site and investigators shall provide all required additional information to the ethics committee and sponsor.

2. Contact person information.

The actual reporting units and contact information at each center location shall prevail.

## XIV. Deviations from Clinical Trial Protocols and Regulations for Clinical Trial Protocol Amendments

**Deviation from the clinical trial protocol**

Refers to situations where the requirements of the clinical trial protocol are not followed, either intentionally or unintentionally.

Program deviations include but are not limited to:

(1) Subjects who did not meet the inclusion criteria were still enrolled in the trial;

(2) The subject did not sign the informed consent form;

(3) Subjects signed the informed consent form only after participating in the trial;

(4) Subjects failed to undergo follow-up as specified in the protocol;

(5) Underreporting or delayed notification of serious adverse events.

**treatment measure**

Any deviations from the protocol should be promptly documented. Clinical trial protocol deviations that affect the rights and interests, safety, and health of participants or the scientific integrity of the clinical trial, including both requested deviations and reported deviations, must be immediately reported by investigators to the medical device clinical trial management department of the clinical trial institution. The department shall then promptly notify the sponsor and report to the ethics committee.

To protect the rights, safety, and health of subjects, deviations occurring in emergency situations that cannot be reported promptly shall be reported in writing as soon as possible after the event in accordance with relevant regulations.

**Clinical Trial Protocol Amendment**

Any revision of the clinical trial protocol during the clinical trial process shall only proceed after obtaining written approval from the ethics committee.

## XV. Direct access to source data and files

At the request of the monitoring officer, ethics committee, or regulatory authority, the investigator/clinical trial institution shall provide access to all trial-related records as required.

Researchers and institutions should allow relevant personnel direct access to raw data to facilitate trial-related monitoring, audits, ethics committee evaluations, and regulatory oversight.

## XVI. Finance and Insurance

Financial and insurance matters will be stipulated in separate agreements.

## XVII. Contents to be included in clinical trial reports

Upon completion of the clinical trial, investigators from each clinical trial site shall separately prepare clinical trial summaries, which shall be submitted to the coordinating investigator for consolidation and completion of the final report after review in accordance with regulations, along with case report forms. Sub-center clinical trial summaries

The report shall at least include an overview of the clinical trial, general clinical data, information description of trial devices, safety and efficacy datasets, incidence rates of adverse events and their management, explanations of protocol deviations, and case report forms.

The clinical trial report shall include summaries of clinical trials from each sub-center.

The clinical trial report shall be consistent with the clinical trial protocol and primarily include the following components: (1) General information; (2) Abstract; (3) Introduction; (4) Clinical trial objectives; (5) Clinical trial methods; (6) Clinical trial content; (7) General clinical data; (8) Investigational medical devices; (9) Statistical analysis methods and evaluation methods employed; (10) Clinical evaluation criteria; (11) Organizational structure of the clinical trial; (12) Ethical considerations; (13) Clinical trial results; (14) Adverse events identified during the clinical trial and their management; (15) Analysis and discussion of clinical trial results, particularly regarding indications, scope of application, contraindications, and precautions; (16) Clinical trial conclusions; (17) Identified issues and improvement recommendations; (18) List of trial personnel; (19) Other relevant information requiring clarification.

The clinical trial report shall be signed by the investigator, dated, reviewed and commented by the medical device clinical trial management department of the clinical trial institution, dated, and stamped with the institution's seal before being submitted to the sponsor.

The clinical trial summary for each sub-center shall be signed by the investigator of that center and dated, reviewed and dated by the medical device clinical trial management department of the center, and stamped with the clinical trial institution seal before being submitted to the lead unit.

## XVIII. Responsibilities Assumed by All Parties

### 1. Sponsor Responsibilities

(1) Initiate, apply for, organize, and monitor clinical trials, and be responsible for the authenticity and reliability of clinical trials.

(2) Organize the development and revision of investigator manuals, clinical trial protocols, informed consent forms, case report forms, relevant standard operating procedures, and other related documents, and be responsible for organizing and conducting necessary training for clinical trials.

(3) The formulation of clinical trial protocols shall not involve exaggerated promotion of the mechanisms and therapeutic efficacy of investigational medical devices.

(4) During the clinical trial process, when the sponsor obtains important information that affects the clinical trial, they should promptly revise the investigator manual and related documents, and submit them to the medical device clinical trial management department of the clinical trial institution for review and approval by the ethics committee.

(5) The sponsor is responsible for the safety of investigational medical devices during clinical trials. If potential risks to subject safety are identified or if trial implementation may alter the ethics committee's approval for continued trial conduct, the sponsor shall immediately notify all clinical trial sites and investigators and take appropriate measures.

(6) If the sponsor decides to suspend or terminate the clinical trial, it shall notify the medical device clinical trial management department of all clinical trial institutions within 5 days and provide a written explanation of the reasons.

(7) The sponsor shall bear the treatment costs and corresponding economic compensation for subjects who suffer injuries or deaths related to clinical trials, except for damages caused by the negligence of medical institutions and their medical staff during diagnosis and treatment activities.

(8) The sponsor shall assume monitoring responsibilities for the clinical trial and select qualified monitors to perform monitoring duties.

(9) To ensure the quality of clinical trials, the sponsor may organize independent evaluators with appropriate training and experience to conduct inspections of the clinical trial implementation process, assessing whether the trial complies with the protocol requirements.
(10) For serious adverse events and device defects that may lead to serious adverse events, the sponsor shall report to the registered regulatory authority and the corresponding health commission within 5 working days upon becoming aware of such incidents. Simultaneously, the sponsor shall notify other clinical trial institutions and investigators involved in the trial, and the medical device clinical trial management department shall promptly inform the ethics committee of the clinical trial institution.
(11) The sponsor shall organize the development of standard operating procedures (SOPs) in accordance with the clinical trial protocol, and conduct training on the clinical trial protocol and the use and maintenance of investigational medical devices for all participating investigators, to ensure consistency in the implementation of the clinical trial protocol and the use of investigational medical devices.
For other matters not covered herein, refer to the sponsor's responsibilities in the Good Clinical Practice for Medical Devices.

**2. Responsibilities of Clinical Trial Institutions and Investigators**

(1) Prior to accepting a clinical trial, the clinical trial institution shall evaluate relevant resources based on the characteristics of the investigational medical device to determine whether to accept the clinical trial.
(2) Clinical trial institutions shall properly preserve clinical trial records and basic documents in accordance with the agreement with the sponsor.

(3) Before the clinical trial, the medical device clinical trial management department of the clinical trial institution should cooperate with the sponsor to submit an application to the ethics committee, and submit the relevant documents as required.

(4) Investigators shall ensure that relevant personnel involved in the trial are familiar with the principles, indications, product performance, operating methods, installation requirements, and technical specifications of the investigational medical device. They must also understand the preclinical research data and safety profiles of the device, and be proficient in risk mitigation strategies and emergency response protocols that may arise during clinical trials.
(5) Investigators shall ensure that all participants in clinical trials fully understand the clinical trial protocol, relevant regulations, characteristics of investigational medical

devices, and responsibilities associated with the clinical trial. They shall also guarantee the enrollment of an adequate number of subjects meeting the protocol inclusion criteria and allocate sufficient time to safely conduct and complete the clinical trial in accordance with regulatory requirements during the agreed trial period.

(6) Investigators shall ensure that the investigational medical devices are used exclusively for the subjects of the clinical trial and shall not charge any fees.

(7) Investigators shall strictly adhere to the clinical trial protocol. Deviations from the protocol or substantial modifications shall not be made without the consent of the sponsor and the ethics committee, or without approval from the National Medical Products Administration (NMPA) as required. However, in emergency situations where subjects are at immediate risk and require immediate intervention, a written report may be submitted retrospectively.

(8) The investigator is responsible for recruiting participants and conducting interviews with participants or their guardians. The investigator has the obligation to provide participants with detailed information regarding the investigational medical device and the clinical trial, inform them of potential benefits and known, foreseeable risks, and obtain a signed and dated informed consent form from the participant or their guardian.

(9) Investigators or other personnel participating in the trial shall not coerce or induce subjects to participate in the trial through improper means.

(10) When investigators identify adverse events beyond the intended scope of the investigational medical device during clinical trials, they shall jointly revise the relevant sections of the informed consent form with the sponsor. The revised document must be submitted to the ethics committee for review and approval in accordance with established procedures, after which the affected subjects or their legal guardians shall re-sign the modified informed consent form to confirm the changes.

(11) Investigators are responsible for making medical decisions related to clinical trials. In the event of clinical trial-related adverse events, the clinical trial site and investigators shall ensure that subjects receive adequate and timely treatment and management. When subjects develop comorbid conditions requiring treatment and management, investigators shall promptly inform the subjects.

(12) In the event of serious adverse events occurring during clinical trials, investigators shall immediately administer appropriate treatment to the subjects.Medical interventions shall be implemented, and a written report shall be submitted to the medical device clinical trial management department of the affiliated clinical trial institution, with written notification to the sponsor. The medical device clinical trial management department shall submit a written

report to the relevant ethics committee and the regulatory authorities of the province, autonomous region, or municipality where the clinical trial institution is located, as well as the National Health Commission (NHC), within 24 hours. For death events, the clinical trial institution and investigators shall provide all required documentation to the ethics committee and the sponsor.

(13) Investigators shall document all adverse events and identified device defects occurring during clinical trials, collaborate with the sponsor to analyze the causes of these events, prepare a written analysis report, and propose recommendations for continuation, suspension, or termination of the trial. The report shall be submitted to the Ethics Committee for review by the medical device clinical trial management department of the clinical trial institution.

(14) Investigators shall ensure that clinical trial data is accurately, completely, clearly, and timely recorded in case report forms. Case report forms shall be signed by the investigator, and any data modifications must be signed and dated by the investigator. Original records shall be retained and remain clearly legible.

(15) Clinical trial institutions and investigators shall ensure the authenticity, accuracy, clarity, and safety of data, documents, and records generated during clinical trials.

(16) Clinical trial institutions and investigators shall be subject to monitoring and verification by the sponsor, as well as oversight by the ethics committee, and shall provide all required records related to the trial. Clinical trial institutions and investigators shall cooperate with inspectors dispatched by regulatory authorities or competent departments of the National Health Commission during inspections.

(17) When clinical trial institutions and investigators identify risks exceeding potential benefits, or have obtained results sufficient to determine the safety and efficacy of the investigational medical device, necessitating suspension or termination of the clinical trial, they shall notify the participants and ensure they receive appropriate treatment and follow-up. Concurrently, detailed written explanations shall be provided in accordance with regulations. If necessary, reports shall be submitted to the regulatory authorities of the respective province, autonomous region, or municipality directly under the central government.

(18) Clinical trial institutions and investigators shall report to the regulatory authorities of the province, autonomous region, or municipality directly under the central government where the sponsor is located, or to the National Medical Products Administration, if the sponsor violates relevant regulations or requests changes to trial data or conclusions.

(19) At the end of the clinical trial, investigators shall ensure the completion of all records and reports. Additionally, investigators must verify that the received investigational medical devices match the quantities used, discarded, or returned, and ensure that any remaining investigational medical devices are properly disposed of and documented for archiving.

(20) Investigators may authorize designated personnel to perform subject recruitment, maintain continuous communication with subjects, record clinical trial data, and manage investigational medical devices as required by the clinical trial. Investigators shall provide relevant training to authorized personnel and establish corresponding documentation.

For other matters not covered herein, refer to the responsibilities of clinical trial institutions and investigators as stipulated in the Good Clinical Practice for Medical Devices.

**The investigator declares that I agree to:**

1. Strictly conduct this clinical trial in accordance with the Helsinki Declaration, current regulations of China, and the requirements of the trial protocol.

2. Accurately record all required data in the Case Report Form (CRF) and complete the clinical trial report on time.

3. The investigational medical devices shall be used exclusively for this clinical trial. During the clinical trial process, the receipt and usage of the investigational medical devices shall be recorded comprehensively and accurately, and the records shall be retained.

4. Allow the sponsor to authorize or dispatch monitors, auditors, and regulatory authorities to conduct monitoring, auditing, and inspection of the clinical trial.

5. Strictly comply with the terms of clinical trial contracts/agreements signed by all parties.

I have read the entire clinical trial protocol, including the aforementioned statements, and I agree to all of the above content.

| |
|---|
| Sponsor's Comment<br><br><br>Signature (Seal)<br>Y M D |

| |
|---|
| Researcher's opinion<br><br><br>Signature<br>Y M D |

| |
|---|
| Opinions from Medical Device Clinical Trial Institutions<br><br><br>Signature (Seal)<br>Y M D |
