# Supplementary material for: LED red light therapy for relief of primary dysmenorrhea: An open, single-group target value clinical trial
Source: PLoS One. 2026 Sep 1;21(9):e0356618. doi: 10.1371/journal.pone.0356618 (PMC13533393; doi:10.1371/journal.pone.0356618)
Supplement: Supplementary file 2 [file pone.0356618.s002.pdf]

医疗器械临床试验方案

方案编号：CYGL-2021-02

评价 LED 红光治疗仪临床应用安全性和有效性的开放、单组目标值临床试验

试验用医疗器械名称：LED 红光治疗仪

型号规格：CY1002C

试验用医疗器械的管理类别：第二类

需进行临床试验审批的第三类医疗器械 是☐ 否☑

中国境内同类产品 有☐ 无☑

方案版本号和日期：v3.0/2022 年 03 月 14 日

临床试验机构：内蒙古科技大学包头医学院第一附属医院

研究者：郭莉

申办者：北京创盈光电医疗科技有限公司

代理人：不适用

填写说明：

1.对于多中心临床试验，封面上的临床试验机构只填写牵头单位，其他机构在方案内容中列出。

2.对于多中心临床试验，封面上的研究者填写协调研究者。

目 录

方案摘要 1
一、申办者信息 4
（一）申办者名称 4
（二）申办者地址 4
（三）申办者联系方式 4
（四）申办者相关资质文件 4
（五）代理人的名称、地址、联系方式及相关资质文件 4
二、多中心临床试验所有临床试验机构和研究者列表 4
三、临床试验目的和内容 4
（一）目的 4
（二）内容 4
四、临床试验的背景资料 4
五、产品的特点、结构组成、工作原理、作用机理与试验范围 6
（一）产品特点 6
（二）产品结构组成、工作原理、作用机理 6
（三）试验范围 6
六、产品的适用范围与禁忌症、注意事项 6
（一）适用范围 6
（二）禁忌症 6
（三）注意事项 7
七、总体设计 7
（一）试验设计 7
1.试验目的 7
2.试验方法选择及其理由 7
3.减少、避免偏倚的措施 7
4.试验用医疗器械 8
5.受试者选择 8
6.有效性评价方法 10
7.安全性评价方法 12

（二）试验流程 12
1.试验流程 12
2.用械规范 14
（三）监查计划 14
八、统计学考虑 15
（一）统计学设计、方法和分析规程 15
（二）样本量的计算 16
1.总样本量 16
2.每病种临床试验例数及其确定理由 18
3.在多中心临床试验中，每个临床试验机构的最低和最高的受试者数量及理由 18
（三）临床试验的显著性水平和把握度 18
（四）预期脱落率 18
（五）临床试验结果的合格/不合格标准 18
（六）基于统计学理由终止试验的标准和理由 19
（七）所有数据的统计方法，连同缺失、未用或错误数据（包括中途退出和撤出）和不合理数据的处理方法 19
（八）报告偏离原定统计计划的程序 19
（九）纳入分析中的受试者的选择标准及理由 19
（十）验证假设时排除特殊信息及其理由（如适用） 19
九、数据管理 20
十、可行性分析 21
（一）成功的可能性分析 21
（二）失败的可能性分析 21
十一、临床试验的质量控制 21
十二、临床试验的伦理问题及知情同意 22
（一）伦理方面的考虑 22
（二）试验方案的审批 22
（三）知情同意过程和知情同意书文本 22

十三、对不良事件和器械缺陷报告的规定 23
（一）不良事件 23
（二）严重不良事件（SAE） 24
（三）器械缺陷 24
（四）报告程序、联络人信息 24
十四、临床试验方案的偏离与临床试验方案修正的规定 25
十五、直接访问源数据、文件 25
十六、财务和保险 25
十七、临床试验报告应涵盖的内容 25
十九、各方承担的职责 26

方案摘要

**医疗器械基本信息：**<br>注册分类：第二类<br>研究器械名称：LED 红光治疗仪<br>型号：CY1002C<br>适用范围：原发性痛经<br>申办者：北京创盈光电医疗科技有限公司
**试验题目：**<br>评价 LED 红光治疗仪临床应用安全性和有效性的开放、单组目标值临床试验
**研究目的：**<br>评价 LED 红光治疗仪用于缓解原发性痛经的安全性和有效性
**研究人群及样本量：**<br>拟选择经确诊的原发性痛经的受试者进行研究。<br>**样本量估算**<br>样本量计算公式：$n_T=\frac{[Z_{1-\alpha}\sqrt{P_0(1-P_0)}+Z_{1-\beta}\sqrt{P_T(1-P_T)}]^2}{(P_T-P_0)^2}$<br>参数设置：<br>1. α，显著性水平：本次试验取 α=0.025（单侧）；<br>2. 1-β，把握度：本次试验 β=0.20；<br>3. P_0 及 P_T：假定试验器械有效率（P_T）为 90%，设定目标值（P_0）为 80%；<br>经计算，样本量为 107 例，考虑 10%的脱落及临床操作，所需样本量为 120 例。
**试验设计**<br>**总体设计：**<br>本试验采用开放、单组目标值设计。<br>**有效性评价指标：**<br>**主要疗效指标：有效率：**根据视觉模拟评分（Visual Analogue Scale，VAS）统计疗效

（评价时间节点：入组后第三次月经期间）

显效：VAS 评分下降率≥70%；

有效：VAS 评分下降率 30%-69%；

无效：VAS 评分下降率≤29%。。

VAS 评分下降率=(（筛选期 VAS 评分-治疗后 VAS 评分）/筛选期 VAS 评分)×100%。

有效=显效+有效。

次要疗效指标：

1. COX 痛经症状量表评分；

2. 受试者使用体验评价（按 5 阶分法计算）：

1）非常不便，不满意，

2）不便，不满意，

3）一般便携，一般满意，

4）便携，满意，

5）非常便携，非常满意。

其中 1）-3）定义为使用体验差，4）-5）定义为使用体验好；

3. 受试者入组后第一次、第二次、第三次月经期间 VAS 评分；

4. 受试者入组后第一次、第二次月经期间有效率。

安全性评价指标：

1. 生命体征

呼吸、脉搏、血压、体温；

2. 不良事件

记录在试验期间发生的任何不良事件，记录不良事件的名称、发生时间、持续时间、严重程度及其与器械的关系；

3. 器械缺陷

指临床试验过程中医疗器械在正常使用情况下存在可能危及人体健康和生命安全的不合理风险，如标签错误、质量问题、故障等。

受试者入选/排除标准：

入选标准：

1) 13-35周岁（含13、35周岁）的女性；

2) 诊断为原发性痛经；

3) 月经周期规律：28±7天；

4) 自测最近2个月经周期的VAS平均疼痛评分值≥4分者；

5) 自愿参加试验，受试者和/或一方监护人自愿签署知情同意书。

排除标准：

1. 经妇科检查及腹部超声证实由于子宫内膜异位症、盆腔炎、子宫腺肌病等妇科疾病或者其他疾病引起的继发性痛经者；
2. 筛选当天1个月内有口服避孕药和/或作用于中枢神经系统性药物和/或接受过痛经相关物理治疗；
3. 筛选当天24 h内使用过镇痛药物；
4. 照射部位皮肤有破溃、感染等异常；
5. 患有严重精神类或其他疾病无法正确行VAS疼痛评分或不能客观描述症状；
6. 既往有光疗过敏史、恶性肿瘤史、系统性红斑狼疮史、出血/溶血性疾病史；
7. 妊娠、哺乳期或入组后3个月内预期妊娠的妇女；
8. 筛选当天1个月内参加过其他医疗器械或药物临床试验者；
9. 研究者认为不适合入组的其他情况，如预计依从性差。

研究期限

自取得伦理批件、完成省局备案且产品到位后 12 个月完成临床试验。

一、申办者信息

（一）申办者名称

北京创盈光电医疗科技有限公司。

（二）申办者地址

北京市北京经济技术开发区运成街 3 号 1 号楼 1 层东侧。

（三）申办者联系方式

何军（13370082505）。

（四）申办者相关资质文件

申办者相关资质文件另附。

（五）代理人的名称、地址、联系方式及相关资质文件

不适用。

二、多中心临床试验所有临床试验机构和研究者列表

临床试验机构和研究者列表见《临床试验总结报告》。

三、临床试验目的和内容

（一）目的

在临床上使用北京创盈光电医疗科技有限公司生产的 LED 红光治疗仪，评价 LED 红光治疗仪用于缓解原发性痛经的安全性和有效性。

（二）内容

本试验采用开放、单组目标值设计。受试者签署知情同意书，符合入组条件后方可入组，使用北京创盈光电医疗科技有限公司生产的 LED 红光治疗仪缓解原发性痛经，从而评价北京创盈光电医疗科技有限公司生产的 LED 红光治疗仪临床应用的安全性和有效性。

四、临床试验的背景资料

原发性痛经，是指无明显生殖器官病变，无明确病因，症状表现为行经期间出现下腹部疼痛、坠胀，并伴有腰酸、恶心、呕吐、头痛等其他不适的一种疾病[1]。原发性痛经是女性青少年常见的主诉，其特点是月经痛和特发性盆腔痛。它影响到 25%的经期妇女，估计患病率为 20-90%，在青春期达到高峰[2]。大约有 15%的女性青少年报告称，她们的原发性痛经非常严重，以至于在每个月经周期中有好几天无法参加正常的社会活动。

原发性痛经的发病因素复杂，月经前子宫内孕酮分泌停止，子宫及血管收缩，引起强烈的炎症反应，导致子宫缺血疼痛，是主要的病因。

在原发性痛经的治疗方式上，包括布洛芬、萘普生钠和甲非那酸在内的非甾体类消炎药是缓解月经疼痛最常用的药物，对原发性痛经有很好的疗效。据报道，口服避孕药对缓解月经疼痛也有效，其可降低在月经期间释放前列腺素、血管加压素及催产素，但因其对机体代谢有明显的影响，因此有更多的不良反应[3]。对于担心不良反应不愿服用非甾体抗炎药或激素治疗的患者，也可以采用非药物镇痛治疗，包括经皮神经电刺激、针灸、远红外、局部热疗和低水平激光治疗。然而，这些治疗方式中几乎没有证据表明哪一种应该是首选的治疗方式[2]。

北京创盈光电医疗科技有限公司生产的LED红光治疗仪（试验器械）由治疗头、输入磁吸座、调节腹带、控制输出连接线、控制器、电源适配器组成，其通过高纯度、高光功率窄波红光，以达到缓解原发性痛经的目的。试验器械主要作用机理为通过光照增加患者体内的环磷酸腺苷（cAMP）和一氧化氮（NO）的浓度，放松子宫平滑肌，增加血液流量，减轻炎症，使平滑肌恢复正常的有节奏的收缩和放松，从而缓解痛经。

试验器械已通过了北京市医疗器械检验所的注册检验，各项指标符合国家标准及产品技术要求的规定。为了确定试验器械在缓解原发性痛经中的安全性及有效性，现根据《医疗器械临床试验质量管理规范》及其它国家相关法规和伦理要求以及该产品的特点和机理，制定本方案进行临床试验。

背景资料参考文献：

[1]张杰,白继庚,白丽霞, 等.特定波长低功率可见光治疗原发性痛经有效性与安全性研究[J].实用妇科内分泌杂志(电子版),2018,5(07):3-7.

[2] Hong G Y , Shin B C , Park S N , et al. Randomized controlled trial of the efficacy and safety of self-adhesive low-level light therapy in women with primary dysmenorrhea[J]. International Journal of Gynaecology & Obstetrics the Official Organ of the International Federation of Gynaecology & Obstetrics, 2016, 133(1):37.

[3]嵇波，任晓暄，赵雅芳,等. 原发性痛经发病机制与防治研究述评[J]. 中国现代医学杂志, 2008, 18(13):4.

五、产品的特点、结构组成、工作原理、作用机理与试验范围

（一）产品特点

LED 红光治疗仪是家用式治疗仪，其采用高纯度、高功率窄波红光，通过光照射达到缓解痛经的目的。

（二）产品结构组成、工作原理、作用机理

1.结构组成

由治疗头、输入磁吸座、调节腹带、控制输出连接线、控制器、电源适配器组成。

2.工作原理与作用机理

长期以来，低强度光疗法(LLLT)一直主要用于缓解患者的肌肉疼痛。LLLT 能引起生物活性：刺激 ATP 合成、细胞代谢、蛋白质合成和细胞增殖、诱导抗炎症反应、刺激神经功能和产生生理活性物质，如一氧化氮(NO)，环磷酸腺苷(cAMP)和内啡肽。

在哺乳动物细胞中，某些颜色的光使细胞内 cAMP 浓度增加。由于细胞内 cAMP 浓度的增加，子宫平滑肌放松。此外，低强度光可以增强 NO 的产生，NO 是一种著名的血管舒张剂和神经递质，可以增加血液流量，减轻炎症。因此，子宫平滑肌的放松可能会使进入平滑肌的血液流量增加。一些受损的平滑肌可以通过增加血液供应获得氧气和营养，使平滑肌恢复正常的有节奏的收缩和放松，从而缓解痛经。

（三）试验范围

确诊为原发性痛经的受试者。

六、产品的适用范围与禁忌症、注意事项

（一）适用范围

原发性痛经。

（二）禁忌症

1. 光敏感者、系统性红斑狼疮；
2. 血压不稳、心率失常、佩戴心脏起搏器；
3. 肿瘤患者；
4. 出血或溶血性疾病患者；
5. 腹部表层急性化脓性炎症患者；
6. 有备孕计划者
7. 其他不适合照射的患者。

（三）注意事项

1. 本产品治疗控制器更换，应在生产厂家指导下进行。

2. 没有生产厂家专业维修经验的人，不得拆卸本产品。

3. 仪器所有部件需要进行维修或装运时，请联系生产厂家。

4. 使用过程中可能会出现月经量增多及例假提前或推迟5-7天，均属正常现象，若出现皮肤干痒、潮红现象必要情况下可自行采取补水保湿处理。

七、总体设计

（一）试验设计

1.试验目的

本试验的目的是评价LED红光治疗仪用于缓解原发性痛经的安全性和有效性。

（1）主要目的

评价LED红光治疗仪的主要临床性能（有效率）是否达到既定的目标值。

（2）次要目的

验证LED红光治疗仪的次要临床性能（COX痛经症状量表评分、受试者使用体验评价、VAS评分）和安全性（生命体征、不良事件）及器械缺陷。

2.试验方法选择及其理由

本试验选择单组目标值方法，理由如下：

1）国内尚无同类产品，故不能实现阳性平行对照。2）目前临床上针对原发性痛经大多数采用心理、药物或手术治疗作为标准治疗，与本项目试验器械的治疗原理及预期目标（使用简单、无损伤、无痛苦、无药物）存在较大的差异，可达到的疗效也存在较大的差异，尚无证据表明哪种方法最优，故不考虑将标准治疗方法作为对照。

故本试验采用单组目标值方法，通过汇总光缓解痛经的文献（见样本量计算依据），将文献可达到的疗效作为本次试验的目标值。

3.减少、避免偏倚的措施

本试验采用单组目标值方法，出现的偏倚为非同期对照偏倚。本试验将制定严格的受试者入排标准、临床常用的疗效评价方法及统一的评价时间节点来控制选择偏倚及评价偏倚。

4.试验用医疗器械

表 1 试验器械基本信息

产品名称	型号	生产单位
LED 红光治疗仪	CY1002C	北京创盈光电医疗科技有限公司

5.受试者选择

（1）入选标准

1) 13-35周岁（含13、35周岁）的女性；

2) 诊断为原发性痛经；

3) 月经周期规律：28±7天；

4) 自测最近2个月经周期的VAS平均疼痛评分值≥4分者；

5) 自愿参加试验，受试者和/或一方监护人自愿签署知情同意书。

（2）排除标准

1) 经妇科检查及腹部超声证实由于子宫内膜异位症、盆腔炎、子宫腺肌病等妇科疾病或者其他疾病引起的继发性痛经者；

2) 筛选当天1个月内有口服避孕药和/或作用于中枢神经系统性药物和/或接受过痛经相关物理治疗；

3) 筛选当天24 h内使用过镇痛药物；

4) 照射部位皮肤有破溃、感染等异常；

5) 患有严重精神类或其他疾病无法正确行VAS疼痛评分或不能客观描述症状；

6) 既往有光疗过敏史、恶性肿瘤史、系统性红斑狼疮史、出血/溶血性疾病史；

7) 妊娠、哺乳期或入组后3个月内预期妊娠的妇女；

8) 筛选当天1个月内参加过其他医疗器械或药物临床试验者；

9) 研究者认为不适合入组的其他情况，如预计依从性差。

（3）剔除标准

1) 违反了重要的入组标准；

2) 未接受治疗者；

3) 试验过程中无任何记录者；

4) 合并使用其他器械而影响疗效判断者；

（4）停止试验的标准

停止试验的情况包括受试者退出试验及试验终止。

退出试验

[1]受试者撤回知情同意书；[2]严重违反临床试验方案；[3]研究者认为不再适合继续进行临床试验者；[4]在临床试验期间妊娠的妇女；[5]受试者死亡；[6]受试者失访；[7]申办者要求终止临床试验；[8]其他退出情况。

退出受试者的处理

[1]所有退出受试者均应保留全部源数据和源文件。当受试者退出后，研究者应采取多种形式如电话、邮件等尽可能与受试者联系，询问理由；[2]将终止临床试验的时间和原因详细记录在病例报告表上；[3]因不良事件而脱落的受试者如最终判断与试验器械存在因果关系，必须记录在病例报告表中，并通知申办者。对因不良事件而终止的受试者必须随访至不良事件得到解决或稳定。

试验终止

指临床试验尚未按方案结束，中途停止全部试验。主要目的是保护受试者权益，保证试验质量。除下述的[5]外，应由申办者和研究者共同协商决定。[1]试验中发生严重安全性问题；[2]试验中发现器械疗效太差，甚至无效，不具临床价值；[3]试验中发现临床试验方案有重大失误，难以评价器械效应；[4]申办者要求终止；[5]国家药品监督管理局因某种原因勒令终止临床试验。

（5）入组时间

受试者签署《知情同意书》后，需完成基线评估，经研究者筛选评估是否满足入选、排除标准，如该受试者可以入组，将采用相应的治疗程序。本试验预计在 2022 年 3 月开始受试者的筛选和入组。

（6）临床试验的预期总体持续时间及其确定理由

临床试验的预期总体时间包含临床试验前的准备、病例入组、研究者检查时间，受试者治疗时间、受试者随访时间、数据统计、总结报告撰写等。受医院门诊量/住院量，受试者依从性等因素的影响，本试验预计临床研究周期（全部病例入组、研究者检查时间，受试者治疗时间、受试者随访时间）为 9 个月，总体持续时间为 12 个月。

（7）每位受试者的预期参与持续时间

本试验包括筛选期、随访期，预计受试者参与持续时间为 3 个月。

（8）临床试验所需的受试者数量

总样本量为 120 例。

6.有效性评价方法

（1）有效性参数的说明

主要评价指标：

有效率：根据视觉模拟评分（Visual Analogue Scale，VAS）统计疗效，将入组后第三次月经期间评价的疗效作为主要评价指标。

次要评价指标

COX 痛经症状量表评分：该量表为痛经评价的专业量表，可用于原发性痛经的疗效评价；

受试者使用体验评价：试验器械为居家使用，受试者独立使用是否具有良好体验是本试验比较关注的方面；

VAS 评分（受试者筛选期、入组后第一次、第二次、第三次月经期间）：VAS 评分是疼痛程度评价应用最广泛的方式；

有效率：受试者入组后第一次、第二次月经期间评价的疗效作为次要评价指标。

（2）评价、记录和分析有效性参数的方法和时间选择

1. 有效率

评价[1]：

显效：VAS 评分下降率≥70%；

有效：VAS 评分下降率 30%-69%；

无效：VAS 评分下降率≤29%。

VAS 评分下降率=((筛选期 VAS 评分-治疗后 VAS 评分)/筛选期 VAS 评分)×100%。

有效=显效+有效。

记录：记录 VAS 评分并计算 VAS 评分下降率，根据评价标准判断相应的疗效，将入组后第一次、第二次月经期间评价的疗效作为次要评价指标，入组后第三次月经期间评价的疗效作为主要评价指标。

分析：按统计分析计划。

时间：筛选期、入组后第一次、第二次、第三次月经期间。

2. COX痛经症状量表评分[2]

评价：由受试者根据《COX痛经症状量表》进行评价并统计分值。

记录：记录COX痛经症状量表评分，症状的严重程度及持续时间分别计分。

分析：按统计分析计划。

时间：筛选期、入组后第一次、第二次、第三次月经期间。

3. 受试者使用体验评价

评价：按5阶分法计算

1）非常不便，不满意，

2）不便，不满意，

3）一般便携，一般满意，

4）便携，满意，

5）非常便携，非常满意。

记录：记录评价的分级。其中1）-3）定义为使用体验差，4）-5）定义为使用体验好。

分析：按统计分析计划。

时间：入组后第一次、第二次、第三次月经期间。

4. VAS评分

评价：在纸上面提供一条10cm的直尺，横线的左端为0，表示无痛；右端为10，表示剧痛；中间部分表示不同程度的疼痛。让受试者根据自我感觉在横线上划一记号，表示疼痛的程度。

记录：研究者根据划定的记号记录VAS分值。

分析：按统计分析计划。

时间：筛选期、入组后第一次、第二次、第三次月经期间。

疗效评价指标参考文献：

[1]范雪莲，薛萍，王良萍．超激光疼痛治疗仪结合中医穴位治疗痛经的疗效观察[J]．世界最新医学信息文摘，2015，000(031):84-85.

[2]马玉侠,马海洋,陈少宗,等.中文版COX痛经症状量表的信效度检验[J].山东

中医药大学学报,2015,39(01):5-7.

7.安全性评价方法

（1）安全性参数的说明

生命体征：呼吸、脉搏、血压、体温。

不良事件：指在临床试验过程中出现的不利的医学事件，无论是否与器械相关。

器械缺陷：指临床试验过程中医疗器械在正常使用情况下存在可能危及人体健康和生命安全的不合理风险，如标签错误、质量问题、故障等。

（2）评价、记录和分析安全性参数的方法和时间选择

1.生命体征

评价：评价受试者相应时间点的生命体征。

记录：记录呼吸、脉搏、血压、体温。

分析：按统计分析计划。

时间：筛选期、入组后第一次、第二次、第三次月经期间。

2. 不良事件

评价：观察是否发生不良事件。

记录：记录不良事件类别、严重程度、因果关系等。

分析：按统计分析计划。

时间：使用器械后至第三次月经期。

3. 器械缺陷

评价：观察是否发生器械缺陷。

记录：记录器械缺陷类别。

分析：按统计分析计划。

时间：使用器械后至第三次月经期。

（二）试验流程

1.试验流程

阶段	筛选期	随访期		
	访视 1	访视 2	访视 3	访视 4
观察时点	-14～0 天	入组后第一次月经期	入组后第二次月经期	入组后第三次月经期
知情同意书	X			

入选/排除标准	X			
基线资料[a]	X			
合并用药[b]	X	X	X	X
既往病史及合并疾病[c]	X			
尿妊娠	X			
生命体征[d]	X	X	X	X
腹部超声及妇科检查	X			
VAS 评分	X	X	X	X
COX 痛经症状量表评分	X	X	X	X
受试者使用体验评价		X	X	X
器械缺陷		X	X	X
不良事件[e]		X	X	X

观测项目及指标：

a) 基线资料包括人口学资料（年龄、身高、体重、出生年月）及自测最近 2 个月经周期的 VAS 平均分值；

b) 记录原发性痛经治疗用药物，如非甾体抗炎药、口服避孕药、钙离子通道阻滞剂；

c) 既往病史记录筛选前 1 年之内的月经疼痛治疗史及光疗过敏史、恶性肿瘤史、系统性红斑狼疮史、出血/溶血性疾病史；

d) 生命体征：呼吸、脉搏、血压、体温；

e) 记录使用器械后发生的不良事件。以下情况可以不用报告严重不良事件：①本身疾病的住院治疗，并不是由于之前发生的新的不良反应事件或原有的疾病恶化：如肿瘤的化疗等；②行政性入住，如年度体检；③与临床反应事件无关的计划自愿性入住，比如进行整容整形手术；④在进入试验前就计划或安排的手术择期入院；⑤非住院部接受当天完成的护理程序，并不涉及住院治疗程序；⑥观察室短期停留；⑦其他，康复中心、护理中心、家庭专业护理。

注：

1）筛选期（-14～0 天）：此期间内进行筛选，筛选合格后待月经来临期间（尚未开始治疗）进行 VAS 评分和 COX 痛经症状量表评分；

2）访视 2-4：持续时间为入组后至受试者第三次月经期所持续的时间，嘱患者入组后第一、二、三次每次来月经前 7 天使用器械（30 分钟/次，1 次/天），月经期间停用，在月经期进行随访；

3）试验期间原则上禁用非留体抗炎药、口服避孕药、钙离子通道阻滞剂、及其他镇痛药物等原发性痛经治疗药物、中枢神经系统性药物，及相关痛经物理治疗。从伦理上考虑，当受试者出现疼痛加剧或难以控制时，允许使用镇痛药物但研究者应予以记录。

2.用械规范

使用流程请详见说明书。

（三）监查计划

1. 监查员要求

监查员应当遵循试验用医疗器械临床试验监查标准操作规程，督促临床试验按照方案实施。

2. 时间安排

监查的次数取决于入组进度，应每月不低于 1 次对临床试验机构做监查。可根据临床实际情况进行调整。

3. 监查实施

（1）在试验前确认临床试验机构已具有适当的条件，包括人员配备与培训符合要求，实验室设备齐全、工作情况良好，预期有足够数量的受试者，参与试验人员熟悉试验要求。

（2）在试验前、中、后期监查临床试验机构和研究者是否遵循有关法规、规范和临床试验方案。

（3）确认每位受试者在参与临床试验前签署知情同意书，了解受试者的入选情况以及试验的进展状况；对研究者未能做到的随访、未进行的试验、未做的检查，以及是否对错误、遗漏做出纠正等，应当清楚、如实记录；对修订的知情同意书，确认未结束临床试验流程并受影响的受试者重新签署。

（4）确认所有病例报告表填写正确，并与原始资料一致；所有错误或者遗漏均已改正或者注明，经研究者签名并注明日期；每一试验的病种、病例总数和病例的性别、年龄、治疗效果等均应当确认并记录。

（5）确认受试者退出临床试验或者不依从知情同意书规定要求的情况记录在案，并与研究者讨论此种情况。

（6）确认所有不良事件、并发症和其他器械缺陷均记录在案，严重不良事件和可能导致严重不良事件的器械缺陷在规定时间内做出报告并记录在案。

（7）监查试验用医疗器械样品的供给、使用、维护以及运输、接收、储存、分发、处理与回收。

（8）监督临床试验过程中相关设备的定期维护和校准。

（9）确保研究者收到的所有临床试验相关文件为最新版本。

（10）每次监查后应当形成书面报告，报告应当包括监查员姓名、监查日期、监查时间、监查地点、监查内容、研究者姓名、项目完成情况、存在的问题、结论以及对错误、遗漏做出的纠正等。

八、统计学考虑

（一）统计学设计、方法和分析规程

1. 一般原则

统计分析将采用 SAS 9.4（或更高版本）统计分析软件。

本次试验统计分析主要为描述性分析。定量指标的描述将计算均值、标准差、中位数、最小值、最大值、下四分位数（Q1）、上四分位数（Q3），分类指标描述各类的例数及百分数。

2. 统计分析计划

1）入选及完成情况

列出各个中心入组及完成病例情况及脱落原因，描述入组病例及安全性、有效性分析数据集的例数。列出未进入 PPS 者的清单。

2）基线分析

描述性分析：计数资料采用频数和构成比描述，计量资料采用均数、标准差、最大值、最小值、中位数及 95%可信区间描述。

基线人口统计学资料分析参照描述性分析方法。

3）有效性分析

主要评价指标

检验假设如下：

H_0：$P_T \leqslant P_0$

H_1：$P_T > P_0$

α=0.025（单侧）。

其中，P_T为主要评价指标的总体参数；P_0为主要评价指标的目标值。

主要评价指标选择的数据集为 FAS 及 PPS 集。

次要指标

次要指标选择的数据集为 FAS 集及 PPS 集，统计分析采用上述一般原则。

4）安全性分析（安全性分析基于 SS 集）

① 生命体征

列表详细描述生命体征，列出异常的项目及所占的百分比。

② 不良事件

试验期间所有不良事件的种类、严重程度及与试验器械的关系等将列表描述。

不良事件将按全部不良事件、相关不良事件分类列表给出，其中，出于保守的考虑，相关不良事件将包括与研究器械“肯定有关”、“很可能有关”、“可能有关”的不良事件。

③ 器械缺陷

试验期间所有器械缺陷等将列表描述。

（二）样本量的计算

1.总样本量

样本量计算公式：$n_T=\frac{[Z_{1-\alpha}\sqrt{P_0（1-P_0）}+Z_{1-\beta}\sqrt{P_T（1-P_T）}]^2}{（P_T-P_0）^2}$

参数设置：1. α，显著水平：本次试验取 α=0.025（单侧）；

2. 1-β，把握度：本次试验 β=0.20；

3. P_0及P_T，被试产品主要评价指标的目标值（P_0）及被试产品主要评价指标的率（P_T）：本次试验以有效率为主要评价指标，假定有效率（P_T）为 90%，本次试验有效率目标值（P_0）为 80%；

经计算，样本量为 107 例，考虑 10%的脱落及临床操作，所需样本量为 120 例。

目标值制定的依据：

目标值设定的依据来源于中英文文献报道的关于光缓解原发性痛经的疗效。

1. 文献[1]报道，特定波长低功率可见光用于缓解原发性痛经的有效率为 76.3%（评价有效的标准：VAS 评分从基线降低大于 10%被认为有效）。

2. 文献[2]报道，研究终点 VAS 评分与基线的 VAS 评分相比，试验组（自粘低功率光）变化为-4.34±2.22 分。该文献只报道了 VAS 分值，未报道有效率。

3. 文献[3-4]报道，在 VAS 10 分的刻度中，有 0.9-1.5 的下降变化可认为有效。

通过文献检索，发现参考价值较大的为文献[1]。本试验定义治疗有效的标准为 VAS 下降率≥30%，高于文献[1]的标准（下降大于 10%），而文献[1]获得的疗效为 76.3%，本试验目标值 80%高于文献[1]获得的疗效，可以认为本试验在疗效评价标准和预计目标均高于文献[1]。

文献[3-4]指出 VAS 变化绝对量 0.9-1.5 为有效，本试验 VAS 评分下降率=(（筛选期 VAS 评分-治疗后 VAS 评分）/筛选期 VAS 评分)×100%。假定入选的受试者治疗前 VAS 评分为 4 分，按照评价标准（VAS 下降率≥30%为有效）其下降至少 1.2 才可评价为有效，根据本试验评价标准列表如下：

基线 VAS 评分	计算公式	最低有效标准	达到最低有效的标准
4	VAS 评分下降率=(（筛选期 VAS 评分-治疗后 VAS 评分）/筛选期 VAS 评分)×100%	VAS 下降率≥30%为有效	下降至少 1.2 才可评价为有效
5			下降至少 1.5 才可评价为有效
6			下降至少 1.8 才可评价为有效
7			下降至少 2.1 才可评价为有效
8			下降至少 2.4 才可评价为有效

可以看出本试验随着基线 VAS 的升高，其评价为有效的标准严格于文献[3-4]定义的有效标准。

故本试验综合考虑文献情况及结合临床专家的意见，将目标值定为 80%。

目标值制定参考文献：

[1] 张杰,白继庚,白丽霞, 等.特定波长低功率可见光治疗原发性痛经有效性与安全性研究[J].实用妇科内分泌杂志(电子版),2018,5(07):3-7.
[2] Hong G Y , Shin B C , Park S N , et al. Randomized controlled trial of the efficacy and safety of self-adhesive low-level light therapy in women with primary dysmenorrhea[J]. International Journal of Gynaecology & Obstetrics the Official Organ of the International Federation of Gynaecology & Obstetrics, 2016, 133(1):37.
[3] Kelly AM (1998) Does the clinically significant difference in visual analogue scale pain scores vary with gender, age, or cause ofpain? Acad Emerg Med 5(11):1086-1090
[4] Abbott J, Hawe J, Srivastava P, Hunter D, Garry R (2001) Intraperitoneal gas drain to reduce pain after laparoscopy: randomized masked trial. Obstet Gynecol 98(1):97-100

2.每病种临床试验例数及其确定理由

本试验入选病种为原发性痛经的受试者，可以认为目前的试验仅针对单一适应症开展，不再对其进行进一步的划分。

3.在多中心临床试验中，每个临床试验机构的最低和最高的受试者数量及理由

本试验将同时在 2 家临床试验机构同时开展，原则上，将尽可能的均匀分配各中心的入组数量（样本量 1：1 分配），以保证充分的中心代表性。但考虑到可行性和入组进度，将会根据实际情况对入组数量进行调整（如采用竞争入组的方式），尽量保证每家中心的入选规模相对平衡。

（三）临床试验的显著性水平和把握度

1. 主要评价指标，显著性水平为 α 为 0.025（单侧），把握度 $1-\beta=0.8$。
2. 其他假设性检验，显著性水平为 $\alpha=0.05$（双侧），把握度 $1-\beta=0.8$。

（四）预期脱落率

预期脱落率不得高于 10%。

（五）临床试验结果的合格/不合格标准

对主要评价指标进行假设检验。当 $P\leq\alpha$ 时拒绝 H_0，认为试验产品达到设计要求。或被试产品主要评价指标 95%置信区间的下限高于目标值，则认为被试产品达到设计要求，临床试验结果合格；反之，临床试验结果不合格。

（六）基于统计学理由终止试验的标准和理由

本试验不设期中分析，不依据统计学理由终止试验。

（七）所有数据的统计方法，连同缺失、未用或错误数据（包括中途退出和撤出）和不合理数据的处理方法

试验开始前制定统计分析计划，数据库锁定后按照统计分析计划进行统计分析编程及逻辑学检测编程，之后进行模拟数据库测试，根据结果出具统计分析报告书。

1.缺失值处理：对缺失数据，将根据所在的数据集划分进行处理；对于主要评价数据，将采用 LOCF 法进行填补，次要评价指标的缺失不作处理。

2.不合理数据数据处理：数据管理过程中，将对数据库中数据进行逻辑性核查，发现不合理数据将采用质疑表（Query）形式向研究者发出疑问，并根据研究者的书面答复对不合理数据进行调整，直至所有不合理数据获得解决，方可锁定数据库。

3.错误数据处理：数据管理过程中，将对数据库中数据进行质量管理，发现错误数据将采用质疑表（Query）形式向研究者发出疑问，并根据研究者的书面答复对错误数据进行更正，直至所有错误数据获得更正，方可锁定数据库。

（八）报告偏离原定统计计划的程序

原则上讲，前述的统计分析一般原则、分析内容和方法、数据处理原则等相关统计分析计划不作重要更改。如需更改，则须经生物统计师、研究者、伦理委员会和申办者共同讨论后确定，并在临床试验方案修改版和临床试验总结报告中说明并给出理由。

（九）纳入分析中的受试者的选择标准及理由

全分析集（Full Analysis Set，FAS）：为尽可能接近于所有受试者的分析集，应包括所有入组且使用过一次器械的受试者。

符合方案集（Per Protocol Set，PPS）：是全分析集的子集，包括已接受方案中规定的治疗、可获得主要评价指标的观察数据、对试验方案没有重大违背的受试者。

安全集（Safety Analysis Set，SS）：包括所有入组且使用过一次器械并进行过安全性评价的受试者。

（十）验证假设时排除特殊信息及其理由（如适用）

无。

九、数据管理

本临床试验的数据管理由数据管理方负责。试验过程中，需确保临床试验数据的真实性、完整性和保密性。数据管理过程应符合医疗器械临床试验管理规定，保证临床试验数据的可溯源性。

（1）数据库制定和管理

根据病例报告表的具体内容，由数据管理人员应用 EpiData 或 EXCEL 等软件对所有的研究指标进行数据编码，制定出数据库，并对数据进行录入、核查和导出。且安排专门人员负责数据库的管理。

（2）原始数据监查

监查员监查试验是否遵循试验方案进行，确认所有病例报告表填写正确完整，并与原始资料完全一致。如有错误和遗漏，及时要求研究者改正。修改时需保持原有记录。清晰可见，改正需经研究者签名并注明日期。病例报告表完成后由监查员及时将病例报告表第 1 联收回并运送至数据管理部门，研究者、监查员及数据管理人员需要对回收的病例报告表进行核对。

（3）数据录入与修改

由数据管理员进行数据录入。

对病例报告表中存在的疑问，数据管理员将发出疑问表（Data Query），并通过监查员向研究者发出询问，研究者应尽快解答并返回，数据管理员根据研究者的回答进行数据确认和修改，必要时可以再次发出疑问表。

（4）数据核查

数据核查包括人工核查、计算机化的程序核查以及数据核查会议。对核查中发现的缺失、异常、逻辑错误等数据问题，数据管理部门应及时出具数据核查报告，递交研究者核实确认后再作更改。

（5）数据库锁定

在临床试验数据录入完成后，由申办者、研究者和数据管理三方共同对数据进行核查，确认数据无误后对数据库进行锁定。随后导出锁定的数据供统计分析使用。

（6）数据存档

在临床试验结束后，数据管理部门应将完整的数据库及相关文件移交给申办

者，由申办者保存至无该医疗器械使用时。

（以上为纸质版病例报告表（CRF）的操作流程，如临床试验中采用 E-CRF，可遵循 E-CRF 相应的数据管理流程。）

十、可行性分析

（一）成功的可能性分析

1.本试验遵守《医疗器械临床试验质量管理规范》、《医疗器械临床试验设计指导原则》等相关法规/规范设计及实施。保证临床试验方案的科学性，也保证临床试验符合监管要求。

2.试验器械原料采用符合国家标准材料，其安全性已得到认可。该器械已通过注册检验，并出具合格报告。器械出厂前已经严格检验，不会存在残次品。

3.本试验过程采用的指标易于观察和记录。

（二）失败的可能性分析

造成治疗失败或效果不良的主要原因可能与下列因素相关：

1.受试器械本身原因对受试者造成伤害；

2.产品出现极特殊的生产瑕疵；

3.受试者依从性差，无法保证有效的试验数据进行统计分析；

4.产品说明书不能有效指导使用者，产品的可操作性差；

除上述原因外，本研究还可能存在其他不可预知的风险，导致研究失败。

十一、临床试验的质量控制

（一）标准操作规程（SOP）培训

制定统一的 SOP，并对所有研究者进行培训。研究者应熟悉掌握临床试验方案的具体实施细则和操作规程，规范记录方式与判断标准。

试验过程中，研究者必须认真执行临床方案，对试验结果有重要影响的各项因素和指标（包括纳入标准、排除标准、有效性评价指标、安全性评价指标等）要进行重点控制，在填写病例报告表时应按填写要求，如实、详细、认真记录病例报告表中各项内容，以确保病例报告表内容完整、真实、可靠。

制定临床试验质量控制相关的标准操作规程，供临床试验机构和研究者遵循。

（二）临床监查

详见监查实施。

十二、临床试验的伦理问题及知情同意

（一）伦理方面的考虑

医疗器械临床试验应当遵循《世界医学大会赫尔辛基宣言》确定的伦理准则。

伦理委员会应当秉承伦理和科学的原则，审查和监督临床试验的实施。

临床试验过程中，如修订临床试验方案以及知情同意书等文件、请求偏离、恢复已暂停临床试验，应当在获得伦理委员会的书面批准后方可继续实施。

伦理委员会应当对本临床试验机构的临床试验进行跟踪监督，发现受试者权益不能得到保障等情形，可以在任何时间书面要求暂停或者终止该项临床试验。

被暂停的临床试验，未经伦理委员会同意，不得恢复。

（二）试验方案的审批

临床试验应当获得医疗器械临床试验机构伦理委员会的同意。在获得伦理委员会批准后方可执行临床试验。

（三）知情同意过程和知情同意书文本

在受试者参与临床试验前，研究者应当充分向受试者和/或无民事行为能力人、限制民事行为能力人的监护人说明临床试验的详细情况，包括已知的、可以预见的风险和可能发生的不良事件等。经充分和详细解释后由受试者和/或其监护人在知情同意书上签署姓名和日期，研究者也需在知情同意书上签署姓名和日期。

知情同意书应当采用受试者和/或监护人能够理解的语言和文字。知情同意书不应当含有会引起受试者放弃合法权益以及免除临床试验机构和研究者、申办者或者其代理人应当负责任的内容。

知情同意书应当注明制定的日期或者修订后版本的日期。如知情同意书在试验过程中有修订，修订版的知情同意书执行前需再次经伦理委员会同意。修订版的知情同意书报临床试验机构后，所有未结束试验流程的受试者如受影响，都应当签署新修订的知情同意书。

知情同意书文本另附。

十三、对不良事件和器械缺陷报告的规定

（一）不良事件

定义

指在临床试验过程中出现的不利的医学事件，无论是否与器械相关。

严重程度判定标准

（1）轻度：询问出；

（2）中度：主动叙述但能忍耐；

（3）重度：有客观表现,难忍耐。

与医疗器械因果关系的判断标准

（1）肯定有关：使用器械及反应发生时间顺序合理；停止使用器械以后反应停止，或迅速减轻或好转；再次使用，反应再现，并可能明显加重；同时有文献资料佐证；并已排除原患疾病等其他混杂因素影响。

（2）很可能有关：无重复使用器械史，余同“肯定”，或虽然有联合使用其他器械或药物，但基本可排除联合使用其他器械或药物导致反应发生的可能性。

（3）可能有关：使用器械与反应发生时间关系密切，同时有文献资料佐证；但引发不良反应的器械或药物不止一种，或原患疾病病情进展因素不能除外。

（4）可能无关：不良反应与使用器械时间相关性不密切，反应表现与已知该器械不良反应不相吻合，原患疾病发展同样可能有类似的临床表现。

（5）无关：不良反应出现不符合试验已知的反应类型；受试者临床状态或其在试验期间采用的治疗方式可能产生该反应，受试者状态改善或停止其它治疗反应消除，重复使用其它治疗方式反应出现。

结局判定

（1）死亡：导致生命结束（须收集死亡原因和死亡时间)；

（2）继续：经治疗后，症状未改善/未恢复；

（3）消失且没有后遗症：经治疗后，症状完全消失，且没有后遗症状；

（4）消失但有后遗症：经治疗后，症状已消失但留有后遗症（记录时应注明后遗症的名称或表现)；

（5）不详：情况不明，未进行跟踪观察、记录，或受试者拒绝。

（二）严重不良事件（SAE）

指临床试验过程中发生的导致：死亡；病人、使用者或他人健康严重恶化，包括：致命的疾病或伤害、身体结构或身体功能的永久性缺陷、需住院治疗或延长住院时间、需要进行医疗或手术介入以避免对身体结构或身体功能造成永久性缺陷；导致胎儿窘迫、胎儿死亡或先天性异常/先天缺损等事件。

（三）器械缺陷

指临床试验过程中医疗器械在正常使用情况下存在可能危及人体健康和生命安全的不合理风险，如标签错误、质量问题、故障等。

可能导致严重不良事件的器械缺陷，研究者应及时告知申办者。申办者应当在获知后 5 个工作日内向所备案的管理部门和同级卫健委主管部门报告，同时应当向参与试验的其他临床试验机构和研究者通报，并经其医疗器械临床试验管理部门及时通知该临床试验机构的伦理委员会。

（四）报告程序、联络人信息

1. 报告程序

在临床试验中出现的所有 SAE，研究者应当立即对受试者采取适当的治疗措施，同时书面报告所属的临床试验机构医疗器械临床试验管理部门，并经其书面通知申办者。医疗器械临床试验管理部门在 24 小时内应当书面报告相应的伦理委员会和临床试验机构所在地省级监管部门。对于死亡事件，临床试验机构和研究者应当向伦理委员会和申办者提供所需要的全部附加资料。

对于 SAE 和可能导致 SAE 的医疗器械缺陷，申办者应当在 5 个工作日内向所备案的监管部门报告，同时应当向参与试验的其他临床试验机构和研究者通报，并经参与试验的临床试验机构的医疗器械临床试验管理部门及时通知该试验机构的伦理委员会。

SAE 的病例报告表应尽可能完整、详细地记录与事件有关的资料。对于死亡事件，临床试验机构和研究者应当向伦理委员会和申办者提供所需要的全部附加资料。

2. 联络人信息。

以各中心所在地实际应报告单位及联系方式为准。

十四、临床试验方案的偏离与临床试验方案修正的规定

临床试验方案的偏离

指有意或者无意地未遵循临床试验方案要求的情形。

方案偏离包括但不限于：

（1）受试者不符合纳入标准，但被纳入试验；

（2）受试者没有签署知情同意书；

（3）受试者在参加试验后才签署知情同意书；

（4）受试者未按方案规定进行随访；

（5）严重不良事件漏报或未及时通报。

处理措施

发生方案偏离应及时记录。影响受试者权益、安全和健康或者临床试验科学性的临床试验方案偏离，包括请求偏离和报告偏离。研究者应当及时向临床试验机构的医疗器械临床试验管理部门报告，并经其及时通报申办者、报告伦理委员会。

为保护受试者权益、安全和健康，在紧急情况下发生的偏离无法及时报告的，应当在事后以书面形式尽快按照相关规定报告。

临床试验方案修正

临床试验过程中修订临床试验方案，应当在获得伦理委员会的书面批准后方可继续实施。

十五、直接访问源数据、文件

根据监查员、伦理委员会或监管部门的要求，研究者/临床试验机构应当提供查阅所需的与试验有关的全部记录。

研究者和机构应允许相关人员直接查阅原始资料，以配合与试验相关的监查、稽查、伦理委员会评估和监管部门。

十六、财务和保险

财务和保险将另制定协议加以规定。

十七、临床试验报告应涵盖的内容

临床试验结束后，各临床试验机构研究者应当分别出具临床试验小结，连同病例报告表按规定经审核后交由协调研究者汇总完成总结报告。分中心临床试验小结

应当至少包括临床试验概况、临床一般资料、试验器械的信息描述、安全性和有效性数据集、不良事件发生率以及处理情况，方案偏离情况说明并附病例报告表。

临床试验报告应当包含各分中心临床试验小结。

临床试验报告应当与临床试验方案一致，主要包括：（1）一般信息；（2）摘要；（3）简介；（4）临床试验目的；（5）临床试验方法；（6）临床试验内容；（7）临床一般资料；（8）试验用医疗器械；（9）所采用的统计分析方法以及评价方法；（10）临床评价标准；（11）临床试验的组织结构；（12）伦理情况说明；（13）临床试验结果；（14）临床试验中发现的不良事件以及其处理情况；（15）临床试验结果分析、讨论，尤其是适应症、适用范围、禁忌症和注意事项；（16）临床试验结论；（17）存在问题以及改进建议；（18）试验人员名单；（19）其他需要说明的情况。

临床试验报告应当由研究者签名、注明日期，经临床试验机构医疗器械临床试验管理部门审核出具意见、注明日期并加盖临床试验机构印章后交申办者。

各分中心临床试验小结应当由该中心的研究者签名并注明日期，经该中心的医疗器械临床试验管理部门审核、注明日期并加盖临床试验机构印章后交牵头单位。

十八、各方承担的职责

1. 申办者职责

（1） 发起、申请、组织、监查临床试验，并对临床试验的真实性、可靠性负责。

（2） 组织制定和修改研究者手册、临床试验方案、知情同意书、病例报告表、有关标准操作规程以及其他相关文件，并负责组织开展临床试验所必需的培训。

（3） 组织临床试验方案的制定中不得夸大宣传试验用医疗器械的机理和疗效。

（4） 在临床试验过程中，申办者得到影响临床试验的重要信息时，应当及时对研

究者手册以及相关文件进行修改，并通过临床试验机构的医疗器械临床试验管理部门提交伦理委员会审查同意。

（5） 申办者对试验用医疗器械在临床试验中的安全性负责。当发现可能影响受试者安全或者试验实施可能改变伦理委员会对继续试验的批准情况时，申办者应当立即通知所有临床试验机构和研究者，并作出相应处理。

（6） 申办者决定暂停或者终止临床试验的，应当在 5 日内通知所有临床试验机构医疗器械临床试验管理部门，并书面说明理由。

（7） 申办者应当为发生与临床试验相关的伤害或者死亡的受试者承担治疗的费用以及相应的经济补偿，但在诊疗活动中由医疗机构及其医务人员过错造成的损害除外。

（8） 申办者应当对临床试验承担监查责任，并选择符合要求的监查员履行监查职责。

（9） 申办者为保证临床试验的质量，可以组织独立于临床试验、并具有相应培训和经验的核查员对临床试验开展情况进行核查，评估临床试验是否符合试验方案的要求。

（10） 对于严重不良事件和可能导致严重不良事件的器械缺陷，申办者应当在获知后 5 个工作日内向所备案的监管部门和同级卫健委报告，同时应当向参与试验的其他临床试验机构和研究者通报，并经其医疗器械临床试验管理部门及时通知该临床试验机构的伦理委员会。

（11） 申办者应当按照临床试验方案组织制定标准操作规程，并组织对参与试验的所有研究者进行临床试验方案和试验用医疗器械使用和维护的培训，确保在临床试验方案执行、试验用医疗器械使用方面的一致性。

其他未尽事宜，参考《医疗器械临床试验质量管理规范》中申办者职责。

2. 临床试验机构和研究者职责

（1） 临床试验机构在接受临床试验前，应当根据试验用医疗器械的特性，对相关资源进行评估，以决定是否接受该临床试验。

（2） 临床试验机构应当按照与申办者的约定妥善保存临床试验记录和基本文件。

（3） 临床试验前，临床试验机构的医疗器械临床试验管理部门应当配合申办者向

伦理委员会提出申请，并按照规定递交相关文件。

（4） 研究者应当确保参与试验的有关工作人员熟悉试验用医疗器械的原理、适用范围、产品性能、操作方法、安装要求以及技术指标，了解该试验用医疗器械的临床前研究资料和安全性资料，掌握临床试验可能产生风险的防范以及紧急处理方法。

（5） 研究者应当保证所有临床试验参与人员充分了解临床试验方案、相关规定、试验用医疗器械特性以及与临床试验相关的职责，并确保有足够数量并符合临床试验方案入选标准的受试者进入临床试验、确保有足够的时间在协议约定的试验期内，按照相关规定安全地实施和完成临床试验。

（6） 研究者应当保证将试验用医疗器械只用于该临床试验的受试者，并不得收取任何费用。

（7） 研究者应当严格遵循临床试验方案，未经申办者和伦理委员会的同意，或者未按照规定经国家药品监督管理局批准，不得偏离方案或者实质性改变方案。但在受试者面临直接危险等需要立即消除的紧急情况下，也可以事后以书面形式报告。

（8） 研究者负责招募受试者、与受试者或者其监护人谈话。研究者有责任向受试者说明试验用医疗器械以及临床试验有关的详细情况，告知受试者可能的受益和已知的、可以预见的风险，并取得受试者或者其监护人签字和注明日期的知情同意书。

（9） 研究者或者参与试验的其他人员，不应当强迫或者以其他不正当方式诱使受试者参加试验。

（10） 研究者在临床试验中发现试验用医疗器械预期以外的不良事件时，应当和申办者共同对知情同意书相关内容进行修改，按照相关工作程序报伦理委员会审查同意后，由受影响的受试者或者其监护人对修改后的知情同意书进行重新签名确认。

（11） 研究者负责作出与临床试验相关的医疗决定，在发生与临床试验相关的不良事件时，临床试验机构和研究者应当保证为受试者提供足够、及时的治疗和处理。当受试者出现并发疾病需要治疗和处理时，研究者应当及时告知受试者。

（12） 在临床试验中出现严重不良事件的，研究者应当立即对受试者采取适当的治

疗措施，同时书面报告所属的临床试验机构医疗器械临床试验管理部门，并经其书面通知申办者。医疗器械临床试验管理部门应当在 24 小时内书面报告相应的伦理委员会以及临床试验机构所在地省、自治区、直辖市监管部门和卫健委。对于死亡事件，临床试验机构和研究者应当向伦理委员会和申办者提供所需要的全部资料。

（13） 研究者应当记录临床试验过程中发生的所有不良事件和发现的器械缺陷，并与申办者共同分析事件原因，形成书面分析报告，提出继续、暂停或者终止试验的意见，经临床试验机构医疗器械临床试验管理部门报伦理委员会审查。

（14） 研究者应当保证将临床试验数据准确、完整、清晰、及时地载入病例报告表。病例报告表由研究者签署姓名，任何数据的更改均应当由研究者签名并标注日期，同时保留原始记录，原始记录应当清晰可辨识。

（15） 临床试验机构和研究者应当确保临床试验所形成数据、文件和记录的真实、准确、清晰、安全。

（16） 临床试验机构和研究者应当接受申办者的监查、核查以及伦理委员会的监督，并提供所需的与试验有关的全部记录。监管部门、卫健委主管部门派检查员开展检查的，临床试验机构和研究者应当予以配合。

（17） 临床试验机构和研究者发现风险超过可能的受益，或者已经得出足以判断试验用医疗器械安全性和有效性的结果等，需要暂停或者终止临床试验时，应当通知受试者，并保证受试者得到适当治疗和随访，同时按照规定报告，提供详细书面解释。必要时，报告所在地省、自治区、直辖市监管部门。

（18） 临床试验机构和研究者对申办者违反有关规定或者要求改变试验数据、结论的，应当向申办者所在地省、自治区、直辖市监管部门或者国家药品监督管理局报告。

（19） 临床试验结束时，研究者应当确保完成各项记录、报告。同时，研究者还应当确保收到的试验用医疗器械与所使用的、废弃的或者返还的数量相符合，确保剩余的试验用医疗器械妥善处理并记录存档。

（20） 研究者可以根据临床试验的需要，授权相应人员进行受试者招募、与受试者持续沟通、临床试验数据记录、试验用医疗器械管理等。研究者应当对其授权的人员进行相关的培训并形成相应的文件。

其他未尽事宜，参考《医疗器械临床试验质量管理规范》中临床试验机构及研究者职责。

研究者声明

我同意：

1.严格按照赫尔辛基宣言、中国现行法规、以及试验方案的要求进行本次临床试验。

2.将所要求的全部数据准确记录于病例报告表(CRF)中,按时完成临床试验报告。

3.试验用医疗器械仅用于本次临床试验，在临床试验过程中完整准确地记录试验用医疗器械的接收和使用情况，并保存记录。

4.允许申办者授权或派遣的监查员、核查员和监管部门对该项临床试验进行监查、核查和检查。

5.严格履行各方签署的临床试验合同/协议条款。

我已全部阅读了临床试验方案，包括以上的声明，我同意以上全部内容。

申办者意见<br><br><br><br>签名（盖章）<br>年　月　日

研究者意见<br><br><br>签名<br>年　月　日

医疗器械临床试验机构意见<br><br><br><br>签名（盖章）<br>年　月　日
