# Supplementary material for: LED red light therapy for relief of primary dysmenorrhea: An open, single-group target value clinical trial
Source: PLoS One. 2026 Sep 1;21(9):e0356618. doi: 10.1371/journal.pone.0356618 (PMC13533393; doi:10.1371/journal.pone.0356618)
Supplement: Supplementary file 3 [file pone.0356618.s003.pdf]

Formal Permission Statement

We, Beijing Charmwin Light Medical Technology Co., Ltd., as the sponsor and legal copyright holder of this clinical trial protocol, hereby grant permission to *PLOS ONE* to publish the full trial protocol alongside the related manuscript.

We confirm that the protocol will be published under the Creative Commons Attribution 4.0 (CC BY 4.0) license, provided appropriate attribution is given.
