# Supplementary material for: LED red light therapy for relief of primary dysmenorrhea: An open, single-group target value clinical trial
Source: PLoS One. 2026 Sep 1;21(9):e0356618. doi: 10.1371/journal.pone.0356618 (PMC13533393; doi:10.1371/journal.pone.0356618)
Supplement: Supplementary file 5 [file pone.0356618.s004.docx]

**Dear Editor,**

Thank you for your review and guidance on our manuscript. We here by briefly explain the items marked as Not Applicable (N/A) in accordance with the TREND Statement Checklist, which are all determined based on the study design of our open, single-group, target value clinical trial and the characteristics of physical intervention, as follows:

1. Item 9 (Blinding): This is an open, single-group clinical trial with a physical intervention (LED red light therapy administered by participants at home independently). Given the intuitive nature of the intervention, blinding of participants and intervention deliverers was not feasible. Additionally, no allocation to multiple study groups was performed, eliminating the premise for the design, implementation and assessment of blinding. Thus, the relevant requirements of this item are not applicable.

2. Item 10 (Unit of Analysis): This study assigned interventions and conducted all statistical analyses at the individual participant level; the unit of assignment was completely consistent with the unit of analysis, with no discrepancy between them. Accordingly, analytical methods for addressing such inconsistencieswere not required, and this relevant requirement is not applicable.

3. Item 18 (Ancillary analyses): This study focused on verifying the overall efficacy and safety of LED red light therapy for primary dysmenorrhea. No subgroup analyses, restricted analyses or other ancillary analyses were prespecified or conducted, and thus no relevant data are available for reporting.

All the above N/A items are highly consistent with our study design, with no omission or incomplete reporting of study content. We have fully reported all applicable items in strict accordance with the TREND Statement guidelines.

Thank you again for your professional guidance. We look forward to your further feedback.
